# Chalcogen Bonds in Selenocysteine Seleninic Acid, a Functional GPx Constituent, and in Other Seleninic or Sulfinic Acid Derivatives

**DOI:** 10.1002/asia.202100545

**Published:** 2021-07-16

**Authors:** Abhishek Tripathi, Andrea Daolio, Andrea Pizzi, Zhifang Guo, David R. Turner, Alberto Baggioli, Antonino Famulari, Glen B. Deacon, Giuseppe Resnati, Harkesh B. Singh

**Affiliations:** ^1^ Department of Chemistry Indian Institute of Technology Bombay Mumbai 400076 India; ^2^ School of Chemistry Monash University Clayton Victoria 3800 Australia; ^3^ IITB-Monash Research Academy Monash University Powai, Mumbai 400076 India; ^4^ Department of Chemistry, Materials and Chemical Engineering “Giulio Natta” Politecnico di Milano Via Luigi Mancinelli 7 20131 Milano Italy

**Keywords:** Selenium, Amino Acids, Chalcogen Bond, Selenocysteine, Sigma-hole

## Abstract

The controlled oxidation reaction of L‐selenocystine under neutral pH conditions affords selenocysteine seleninic acid (3‐selenino‐L‐alanine) which is characterized also by means of single‐crystal X‐ray diffraction. This technique shows that selenium forms three chalcogen bonds (ChBs), one of them being outstandingly short. A survey of seleninic acid derivatives in the Cambridge Structural Database (CSD) confirms that the C−Se(=O)O− functionality tends to act as a ChB donor robust enough to systematically influence the interactional landscape in the solid. Quantum Theory of Atom in Molecules (QTAIM) analysis proves the attractive nature of the short contacts observed in crystals containing the seleninic functionality and calculation of surface molecular electrostatic potential (MEP) reveals that remarkably positive σ‐holes can frequently be found opposite to the covalent bonds at selenium. Both CSD searches and QTAIM and MEP approaches show that also the sulfinic acid moiety can function as a ChB donor, albeit less frequently than the seleninic acid one. These findings may contribute to a better understanding, at the atomic level, of the mechanism of action of the enzymes that control oxidative stress and ROS deactivation and that contain selenocysteine seleninic acid and cysteine sulfinic acid in the active site.

## Graphical abstract

**L‐selenocysteine seleninic acid displays in the solid state** three short chalcogen bonds. Analyses of the Cambridge Structural Database and theoretical studies prove that seleninic acid derivatives frequently show the presence of positive σ‐holes and function as robust chalcogen bond donors. Sulfinic acid derivatives behave similarly, but σ‐holes are less positive and chalcogen bonds are formed less commonly.

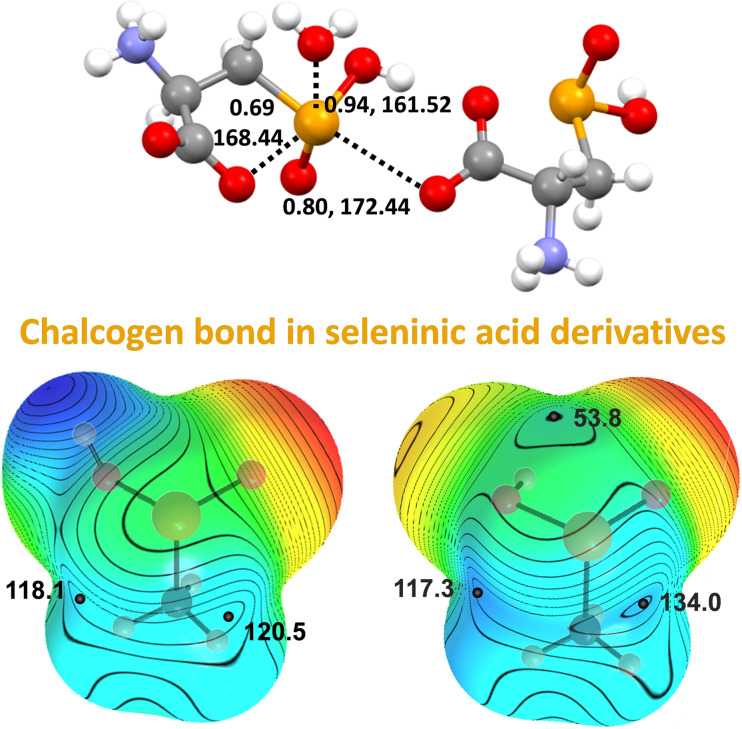

## Introduction

**Scheme 1:**
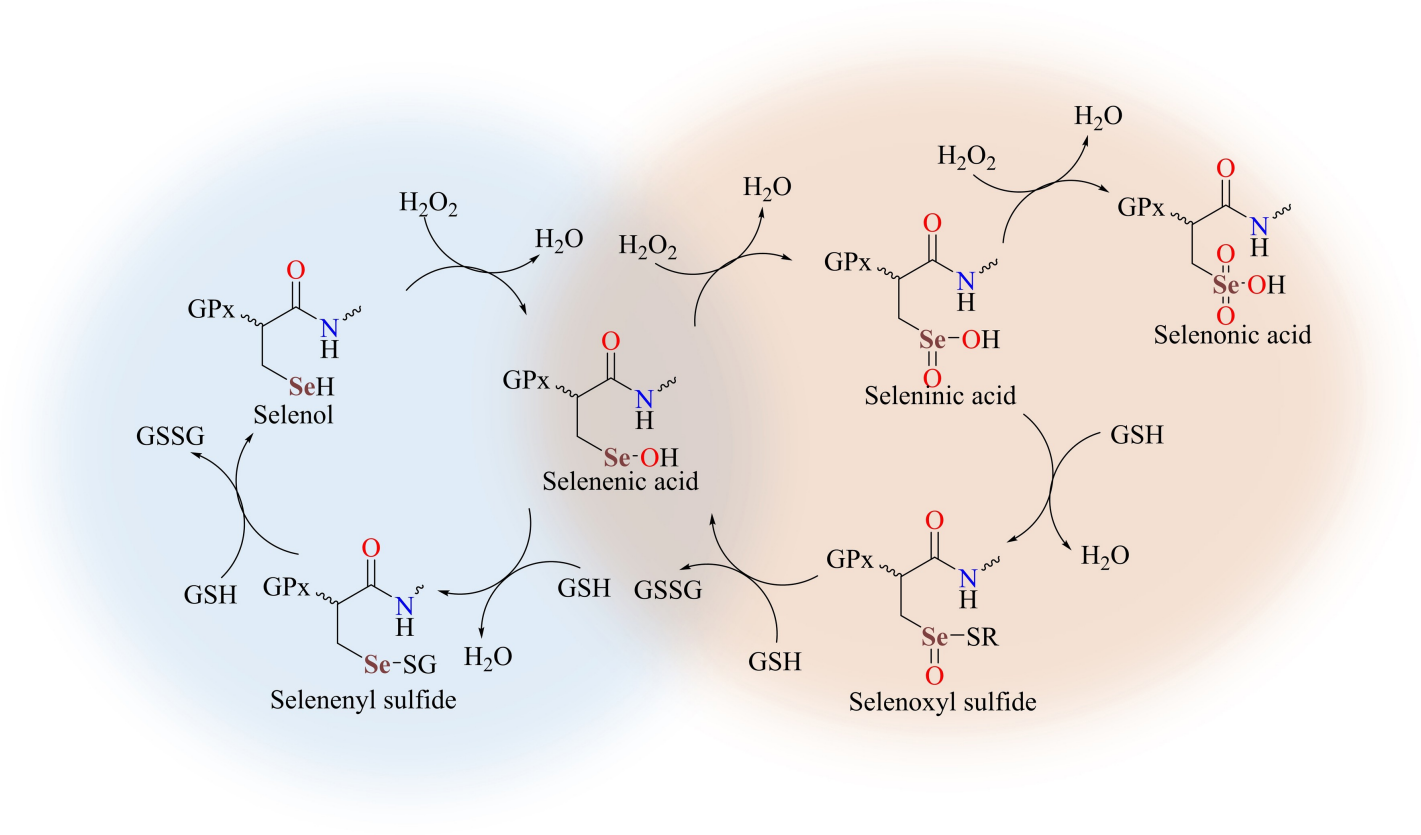
Proposed mechanism for the catalytic activity of GPx enzyme.^15–18^ The GPx main cycle is highlighted in light blue.

The 21^st^ genetically encoded amino acid, selenocysteine [HSeCH_2_CH(NH_2_)COOH] shows specific redox properties involving the selenol group (−SeH) and this behavior leads to the catalytic advantages it offers in biological systems as an antioxidant.^[1]^ *Glutathione peroxidase* (GPx)[^2^, ^3^, ^4^] is a selenoenzyme which contains, at the active site, multiple selenocysteine units and catalyzes the reduction of harmful hydro/organic peroxides into water/alcohols thus protecting the cellular constituents from oxidative stress[^5^, ^6^, ^7^] caused by reactive oxygen species (ROS). Various organic selenides, diselenides, ebselen (2‐phenyl‐1,2‐benzoselenazol‐3‐one) or its derivatives, and the reduced form of selenosubtilisin, a semisynthetic selenoprotein, are important mimics of the GPx enzymatic activity.[^8^, ^9^, ^10^, ^11^] Among these, ebselen is the most studied synthetic mimic due to its very low toxicity and weak Se−N bond^[12]^ which contribute to its effective GPx‐like antioxidant activity in physiological systems.[^13^, ^14^] The anti ROS activity of GPx occurs *via* a catalytic cycle that involves the oxidation of the selenol moiety (E−SeH) (E=enzyme) by peroxides (H_2_O_2_ in Scheme 1) to generate the selenenic acid (E−SeOH) which can react with the glutathione cofactor (GSH) yielding a reactive intermediate selenenyl sulfide (E−SeSG). The catalytic cycle of GPx enzyme is completed by a second GSH molecule that attacks the selenenyl sulfide (E−SeSG) intermediate to regenerate the active selenol (E−SeH), with the release of oxidized cofactor GSSG.[^15^, ^16^, ^17^, ^18^] Alternatively, at higher concentrations of active peroxides a deviation occurs from the primary catalytic cycle of GPx and the intermediate selenenic acid (E–SeOH) undergoes oxidation to the corresponding seleninic acid (E−SeO_2_H) and further on to the selenonic acid (E−SeO_3_H) (Scheme 1).

An investigation of the mechanism of the enzymatic activity indicates that the reduction of the seleninic acid to corresponding selenenic acid by thiol cofactors like GSH is very fast and leads back to the main catalytic cycle of GPx enzyme.[^19^, ^20^, ^21^] Structural evidence of this alternative catalytic path comes from the crystal structure of a human GPx isoform (GPx4),^[22]^ displaying the selenium atom in the oxidation state of seleninic acid. Moreover, G. Mugesh and co‐workers have successfully monitored the formation of seleninic acid within living cells using an ebselen‐based fluorescent probe.^[23]^ The reported potential use of synthetic seleninic acid derivatives in enzymatic activity and inhibition[^24^, ^25^] confirms the biological relevance of these oxidized forms of selenium. For instance, methaneseleninic acid (CH_3_SeO_2_H) has been reported to exert a remarkable anticancer activity, inducing the apoptosis of liver cancer cells upon reaction with intracellular GSH^[26]^ and inhibiting angiogenesis by down‐regulating the expression of integrin β3.^[27]^

**Scheme 2:**
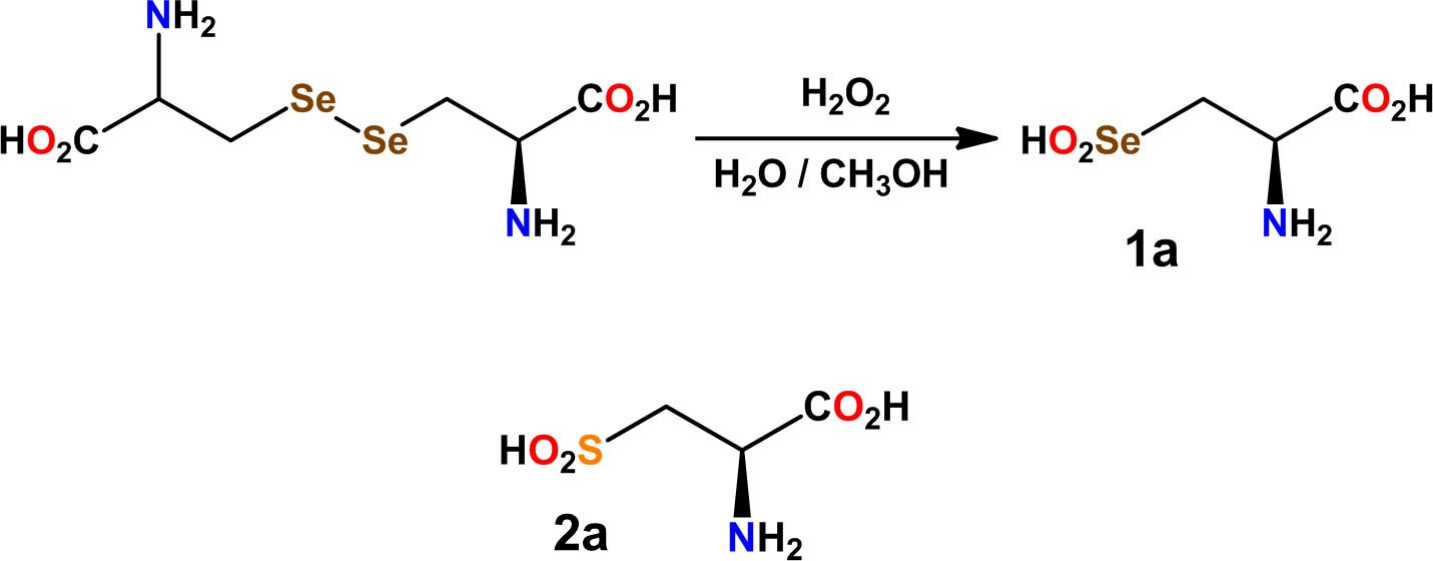
Synthesis of L‐selenocysteine seleninic acid (**1** **a**) and molecular structure of L‐cysteine sulfinic acid (**2** **a**).

The Protein Data Bank (PDB)^[28]^ contains some examples of biomolecules comprising the residue of selenocysteine seleninic acid (hereinafter selenocystinic acid, **1** **a**, Scheme 2). Detailed information on the structural and interactional features of selenocystinic acid free from possible constrains of the protein environment might help in understanding specific functional aspects of these biomolecules, even more as the selenium role in the GPx catalytic pathway may be regarded as a consequence of the propensity of selenium to act as electrophilic species.^[29]^ However, to the best of our knowledge, no structural characterization of **1** **a** is reported.

In this paper we describe the single crystal X‐ray structure of the selenocystinic acid (**1** **a**) (obtained through oxidation of L‐selenocystine with hydrogen peroxide, Scheme 2).[^30^, ^31^] We show that the compound forms three Se**⋅⋅⋅**O short contacts^[32]^ that seemingly affect the conformation and the overall packing in the crystal and can be rationalized as chalcogen bonds (ChBs) thanks to their directionality.^[33]^ The ChB is the non‐covalent interaction wherein an element of group 16 of the periodic table functions as the electrophilic site.^[34]^ The ChB belongs to the larger set of the σ‐hole interactions,^[35]^ namely the weak bondings wherein an electron rich species (*e*. *g*., an anion, a lone pair possessing atom, a double bond, or an aromatic system) gives rise to directional short contacts as they attractively interact with the region of most positive electrostatic potential (σ‐hole) which can be located on an atom opposite to a single covalent bond formed by the atom. A Quantum Theory of Atom in Molecules (QTAIM)^[36]^ study of the Se**⋅⋅⋅**O short contacts in **1** **a** reveals the presence of bond critical points (BCP), namely proves the attractive nature of these interactions. An analysis of the Cambridge Structural Database (CSD) suggests that in the solid the formation of ChBs by the selenium atom of seleninic acid derivatives is quite common. The computation of the molecular electrostatic potential (MEP) on the surface of these derivatives frequently shows the presence of σ‐holes on selenium at the expected positions. Interestingly, CSD inspection and MEP analyses show that also the sulfur atom of sulfinic acid derivatives can function as ChBs donor, although this tendency is less pronounced than that of selenium in seleninic acid derivatives. Nevertheless, the relevance of the ChB donor ability of the sulfinic acid moiety may be even more impacting than that of the seleninic acid moiety due to the more common occurrence and greater functional importance of sulfur and its derivatives. For instance, sulfinic esters are key intermediates in the synthesis of chiral sulfoxides.^[37]^ In relation to the topics discussed above, we may also mention that cysteine sulfinic acid (**2** **a**, Scheme 2) is involved *in* *vivo* in redox processes regulation.[^38^, ^39^] Specifically, the cysteine residues in the active site of *peroxiredoxins* (Prx) enzymes are oxidized to corresponding sulfinic acids by peroxides and other ROS in order to relieve the oxidative stress in cells.[^40^, ^41^, ^42^] In mammalian cells, *sulfiredoxin* enzymes selectively reduce the inactive sulfinic form of Prx in order to repair oxidatively damaged proteins in a redox cycle similar to that of GPx.

## Results and Discussion

In order to synthesize L‐selenocysteine seleninic acid (**1** **a**), the precursor L‐selenocystine was obtained from β‐chloro‐L‐alanine following the procedure reported by Alewood and co‐workers (Scheme 2).^[43]^ The controlled oxidation of selenocystine was achieved by using H_2_O_2_ (4 eq.) at 10 °C in a 4 : 1 methanol/water mixture under pH conditions close to physiological ones.

In the ^77^Se NMR spectrum of **1** **a**, recorded in CD_3_OD, a single resonance was observed at 1221 ppm which is within the range of chemical shift values (δ, 1240–1175) observed for other seleninic acids.^[10]^ For example, Satheeshkumar *et* *al*. and Block *et* *al*. recorded the ^77^Se NMR spectrum of **1** **a** at different pH conditions and found signals at 1207 and 1195 ppm.[^30^, ^31^] In the FT‐IR spectrum of **1** **a**, the sharp peaks at 1593 and 1343 cm^−1^ correspond to the asymmetric and symmetric stretching of carboxylate group, respectively. The intense peaks at 782 and 886 cm^−1^ are attributed to the Se−O stretching frequencies.

The selenocystinic acid (**1** **a**) was unambiguously characterized by single crystal X‐ray diffraction. The compound crystallized as a monohydrate species in the chiral orthorhombic space group *P2_1_2_1_2_1_*. In the asymmetric unit there is a single amino acid molecule which is in the form of carboxylate zwitterion (HO_2_SeCH_2_CH(NH_3_^+^)COO^−^) while selenocysteic acid crystallizes as a selenonate zwitterion (^−^O_3_SeCH_2_CH(NH_3_^+^)COOH).^[30]^ From the molecular point of view, the geometry around the Se atom in **1** **a** is a distorted tetrahedron as the selenium lone pair is stereochemically relevant (O−Se−O angle is 101.41° and O−Se−C angles are 87.71° and 89.68°). The O−Se−O angle is fairly similar to the corresponding angle in methaneseleninic acid (103.51°)^[44]^ and in between analogous angles in sulfinic and tellurinic acids (e. g., 107.50(7)° in methanesulfinic acid^[45]^ and 96.00(1)° in pyridinetellurinic acid^[46]^). The Se−C−C−COO torsional angle for **1** **a** is 32.3°, a value much smaller than that of selenocysteic acid (52.2°),^[30]^ possibly due to the greater steric hindrance of the sulfonate moiety.

The two selenium bonded oxygen atoms are different, no chirality inversion or proton shift occur in solid **1** **a**, and the selenium atom is chiral and enantiopure, the absolute configuration being (*S*) only. The isolation of the **1** **a** diastereoisomer having the (2*R*,*S*_Se_) configuration does not imply a diastereoselective oxidation. Optically active seleninic acids racemize in solution^[44]^ and the exclusive isolation of the crystalline (2*R*,*S*_Se_) diastereoisomer may occur also from solutions containing the (2*R*,*S*_Se_) and (2*R*,*R*_Se_) diastereoisomers through a process of continuous precipitation of the (2*R*,*S*_Se_) isomer and its reinstatement in solution via racemization at selenium. The single/double bond notation is adopted in this manuscript to designate the two selenium‐oxygen bonds and in **1** **a** the Se–OH and the Se=O bonds separations are 178.6 and 167.8 pm, respectively. These values are in good agreement with the typical selenium‐oxygen bonds separations in seleninic acids,[^44^, ^47^] are significantly longer than those in sulfinic acids (e. g., 159.5 and 149.5 pm in methanesulfinic acid),^[45]^ and are shorter than those in tellurinic acids (e. g., 196.6 and 183.4 pm in pyridinetellurinic acid),^[46]^ coherent with the different size of the chalcogen atoms (Table 1S). Selenium‐oxygen distances are longer in **1** **a** than the corresponding distances in selenocysteic acid (164.1–163.0 pm).

**Table 1:** Geometric parameters (distances and angles) of ChBs found in exemplary seleninic acid derivatives from the CSD. V_s,max_ features of the same compounds (resulting from the MEP at 0.001 a.u. isosurface) are also reported along with V_S,max_ angular position.

Compound (Refcode)	Se⋅⋅⋅O separation (pm), respective Nc [O/C−Se⋅⋅⋅O angle (°)]				V_S,max_ (kJ/mol) on Se bond extension [O/C−Se⋅⋅⋅V_S,max_ angle (°)]		
	O=Se⋅⋅⋅O	O−Se⋅⋅⋅O	C−Se⋅⋅⋅O		O=Se⋅⋅⋅V_S,max_	O−Se⋅⋅⋅V_S,max_	C−Se⋅⋅⋅ V_S,max_
**1** **b** ^[44]^ (AWAVOJ)	331.3, 0.97 [174.26]	315.6, 0.92 [175.42]	^[a]^		118.1 [*164*]	120.5 [*146*]	^[b]^
**1** **c** ^[52]^ (QOLHAC)	^[a]^	274.9, 0.80 [163.35]	290.7, 0.85 [176.78]		^[b]^	102.3 [*166*]	^[b]^
**1** **d** ^[53]^ (BATFAD)	^[a]^	302.7, 0.88 [157.78]	292.3, 0.85 [174.70]		^[b]^	134.6 [*159*]	^[b]^
**1** **e** ^[54]^ (SUMCIN)	^[a]^	294.6, 0.86 [177.51]	318.2, 0.93 [167.85]		104.1 [*173*]	99.3 [*160*]	43.9 [*169*]
**1** **f** ^[55]^ (LAYFAT)	329.4, 0.96 [177.12]	276.0, 0.81 [162.84]	^[a]^		^[b]^	131.2 [*147*]	45.1 [*158*]
**1** **g** ^[56]^ (ISUJEM)	326.2, 0.95 [160.45]	274.7, 0.80 [151.90]	313.0, 0.91 [169.84]		104.1 [*160*]	^[c]^	60.9 [*157*]
**1** **h** ^[57]^ (GEKWIC)	303.8, 0.89 [148.16]	262.8, 0.77 [154.83]	295.0, 0.84 [161.57]		169.4 [*169*]	^[c]^	107.4 [*158*]
**1** **i** ^[58]^ (TESEAN)	310.8, 0.91 [157.69]	270.1, 0.79 [159.25]	^[a]^		^[b]^	152.7 [*148*]	61.2 [*163*]
**1** **j** ^[59]^ (XEHHIB)	278.6, 0.76 [170.03]	271.4, 0.74 [173.26]	319.2, 0.87 [155.35]		^[b,d]^	568.2^[e]^ [*174*]	^[b]^

[a] No ChB on the approximate elongation of the considered covalent bond. [b] No V_S,max_ on the approximate elongation of the considered covalent bond. [c] No V_S,max_ is detected probably due to the presence of the intramolecular O−S⋅⋅⋅ON(O) ChB. [d] The O=Se moiety is protonated, i. e., a −Se(OH)_2_^+^group is present rather than a −Se(=O)OH group; a V_S,max_ is found on the elongation of only one Se–OH covalent bond due to the different orientation of the two Se−OH groups with respect to the phenyl ring. [e] The large and positive V_S,max_ value is related to the cationic nature of the molecule.

**Figure 1:**
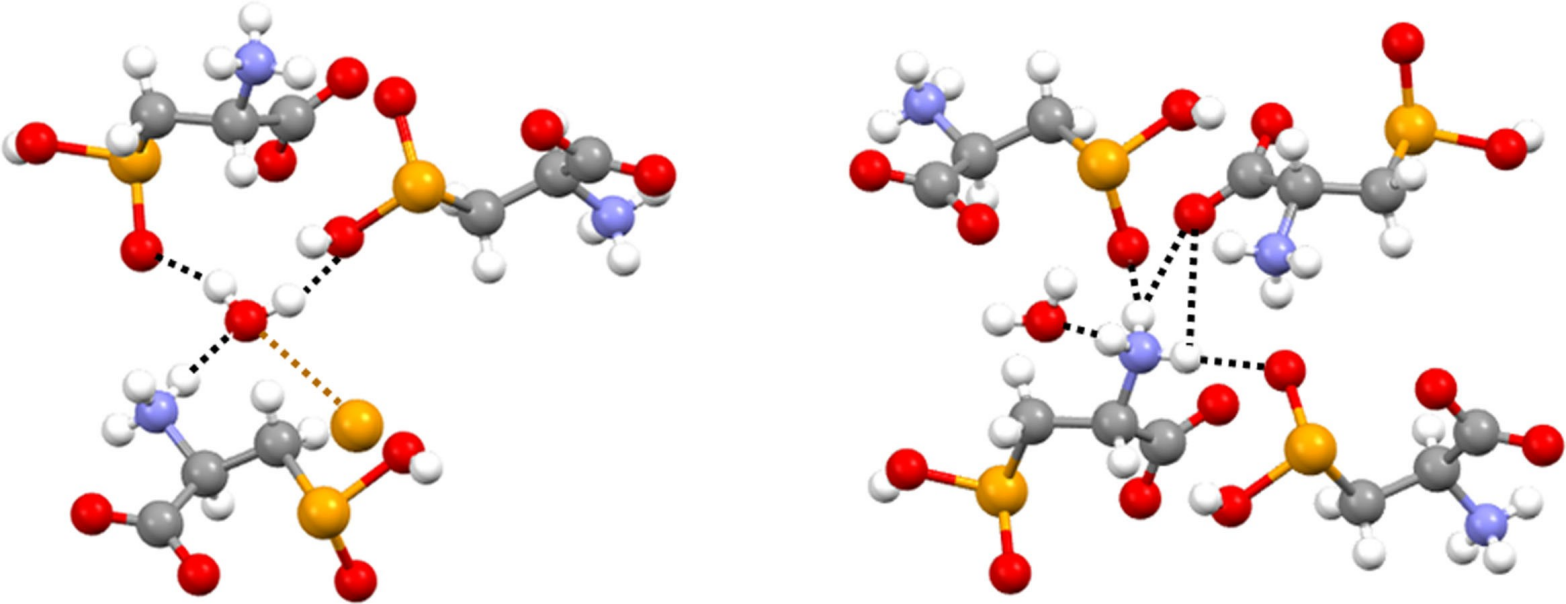
Representation (Mercury 4.3.1) of the network of: (left), HBs (black dotted lines) and ChB (ocher dotted line) formed by a crystallization water molecule; (right), HBs (black dotted lines) formed by an ammonium functionality. Color codes: gray, carbon; whitish, hydrogen; red, oxygen; blue, nitrogen; ocher, selenium.

**Figure 2:**
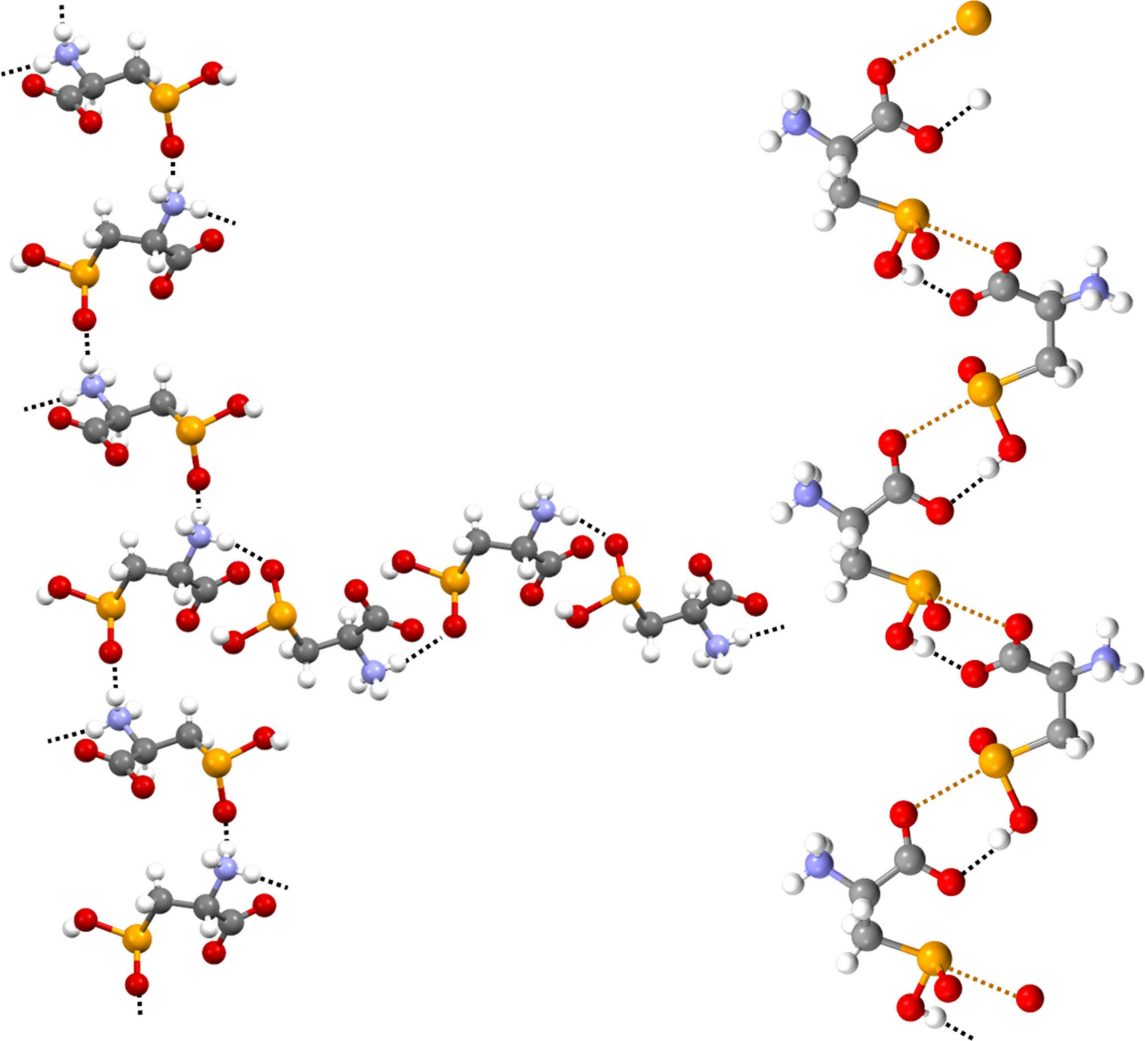
Partial representation (Mercury 4.3.1) of the infinite chains formed by: (left), two geminal ammonium hydrogen atoms linked via two N−H**⋅⋅⋅**O=Se HBs (black dotted lines) to the seleninic acid oxygen atoms of two different selenocystinic acid molecules; (right), the double linking of seleninic acid and carboxylate functionalities *via* a SeO−H**⋅⋅⋅**OC HB (black dotted line) and a C−Se**⋅⋅⋅**OC ChB (ocher dotted line). Color codes: gray, carbon; whitish, hydrogen; red, oxygen; blue, nitrogen; ocher, selenium.

From the supramolecular point of view, the electrostatic attraction between cationic and anionic sites is probably a major driving force in the packing. The interactional landscape in the crystal is heavily affected by the presence of the dense net of hydrogen bonds (HBs) associated with the ammonium carboxylate zwitterion array. The hydration water molecule is pinned in its position by three HBs as it acts as a bidentate donor and monodentate acceptor. The two hydrogen atoms form two short contacts with the oxygen atoms of two different seleninic acids and the oxygen forms one short contact with an ammonium hydrogen (Figure 1, left). The N**⋅⋅⋅**O distance is 282.9 pm, a separation typical for charge‐assisted HBs.[^48^, ^49^] The two other ammonium hydrogen atoms form two HBs each: one is an N−H**⋅⋅⋅**O=Se close contact with a seleninic oxygen atom of two different **1** **a** molecules and the other is an N−H**⋅⋅⋅**OC short contact with the same carboxylic oxygen atom of a third **1** **a** molecule (Figure 1, right). The two N−H**⋅⋅⋅**O=Se interactions give rise to two infinite and nearly orthogonal chains in which the N**⋅⋅⋅**O distances are 280.4 and 284.0 pm (Figure 2, left). The carboxylate anion acts as tridentate HB acceptor. One oxygen forms two intermolecular N−H**⋅⋅⋅**OC HBs with two hydrogen atoms of an ammonium group as discussed above (Figure 1, right). The other oxygen forms one SeO−H**⋅⋅⋅**OC intermolecular contact (O**⋅⋅⋅**O distance is 258.4 pm) which gives rise to an infinite chain along which adjacent selenocystinic acids are connected through a supramolecular synthon that recalls, from the geometric point of view, the carboxylic acid dimer.^[50]^ In fact, the seleninic acid and the carboxylate moieties are double linked by the SeO−H**⋅⋅⋅**OC HB mentioned above and also a C−Se**⋅⋅⋅**OC ChB (Figure 2, right).

**Figure 3:**
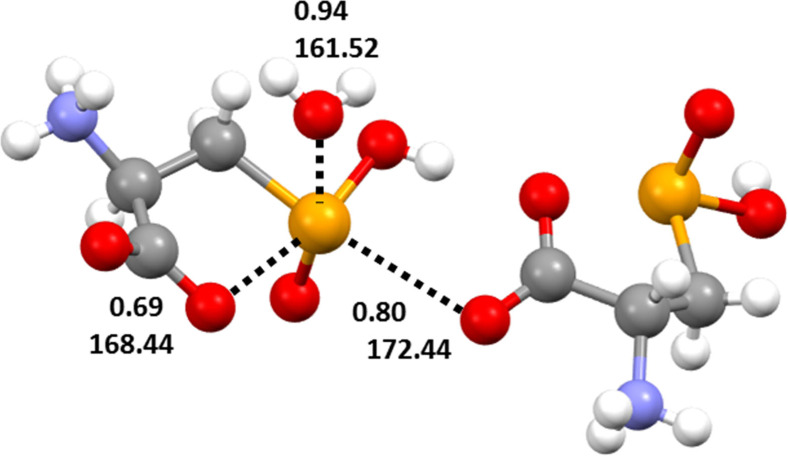
Representation (Mercury 4.3.1) of the three ChBs formed by selenium in **1** **a**. Nc values^[51]^ and angles (°) of the interactions are reported. Color codes: gray, carbon; whitish, hydrogen; red, oxygen; blue, nitrogen; ocher, selenium.

Despite the multiple pinning of **1** **a** within this tight web of HBs and the resulting constraints, the selenium atom forms all the three possible ChBs (Figure 3). The shortest Se**⋅⋅⋅**O interaction is the intramolecular contact formed opposite to the HO–Se covalent bond by one of carboxylate oxygen atoms. The O−Se**⋅⋅⋅**O angle is 168.44° and the Se**⋅⋅⋅**O separation is 251.7 pm, corresponding to a normalized contact (Nc)^[51]^ of 0.69 (Figure 3). This ChB is probably so close due to its intramolecular nature and the strong electron donor ability of the anionic oxygen. The longest Se**⋅⋅⋅**O ChB is the O=Se**⋅⋅⋅**OH_2_ contact the Nc value of which is nevertheless 0.94, a surprisingly small number if considered that the water oxygen may be a poor donor of electron density as involved also in an HB with an ammonium hydrogen (Figure 1). The ChB opposite to the methylene group is formed by a carboxylate oxygen. The interaction is the closest to linearity (the C−Se**⋅⋅⋅**O angle is 172.44°) and its separation is in between those described above (Nc is 0.80). These data prove that the overall crystal packing of **1** **a** is determined by a notable synergy between HBs and ChBs and suggest that the selenium atom in solid **1** **a** is a robust ChB donor.

**Scheme 3:**
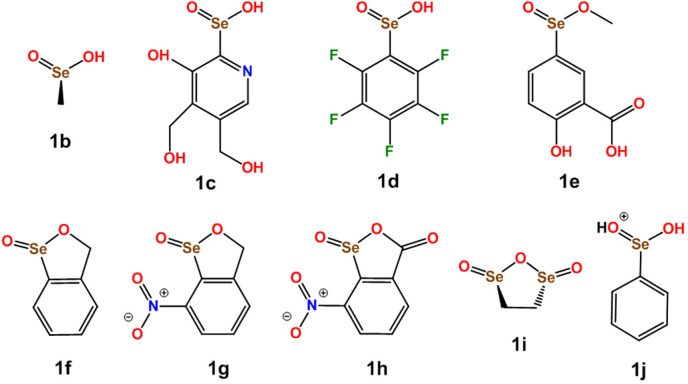
Chemical structures of the seleninic acids reported in Table 1.

**Figure 4:**
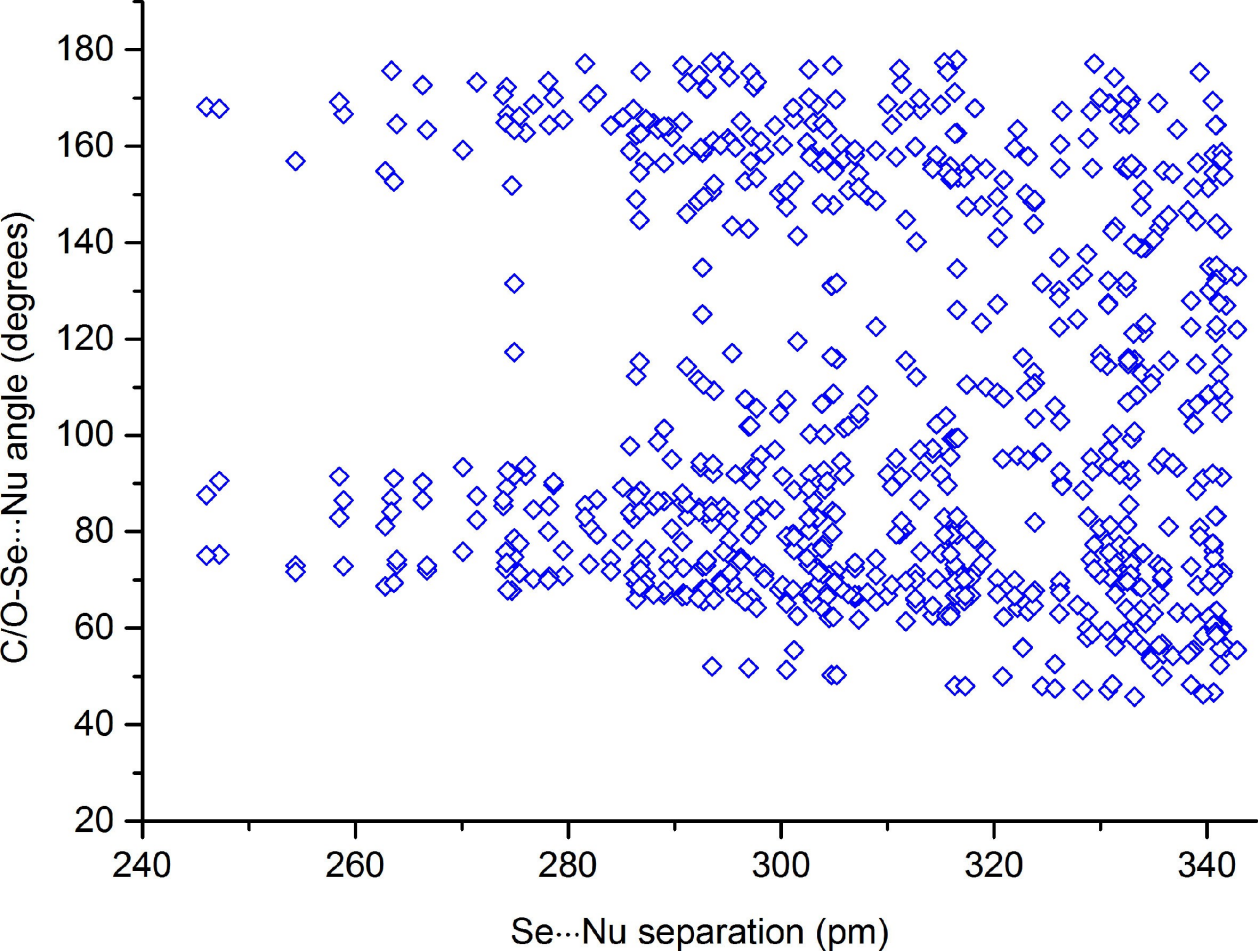
Scatterplot of C/O−Se**⋅⋅⋅**Nucleophile ChBs versus Se**⋅⋅⋅**Nucleophile distances for structures present in the CSD. See caption of Table 2S for criteria used to identify ChBs in CSD.

A survey of the CSD was performed to assess how common the tendency of selenium in seleninic acid derivatives to function as ChB donor is. Table 2S lists all compounds in CSD wherein a ChB appears and Table 1 itemizes the interaction separations and angles of the exemplary structures sketched in Scheme 3. The number of compounds showing the ChB presence in the CSD is large enough to allow for some general trends to be reliably identified. ChBs are found in crystals of seleninic acids and various functionalities thereof (*e*. *g*., esters,[^55^, ^56^] anhydrides,[^57^, ^58^] and salts^[60]^). The seleninic acid moiety can act as multidentate ChB donor and form short and directional contacts also when in compounds as polyfunctional as vitamin B6 analogue **1** **c**. Both anionic and neutral atoms (*e*. *g*., oxygens of sulfonate,^[59]^ nitrate,^[60]^ carbonyl,[^54^, ^57^] or water^[53]^ groups) can function as donors of electron density. The formation of all the three possible ChBs is occasional,^[61]^ at least one or two interactions are present in nearly two third or one fourth of the structures (Table 2S), so that the general ability of seleninic functionality to be a fairly robust ChB donor moiety in the solid is proven. The site opposite to the O−Se covalent bond is more prone to attractively interact with the donor of electron density than the sites opposite to the O=Se and C−Se bonds (Table 3S). Similar to other σ‐hole interactions,[^33^, ^62^] shorter contacts tend to be closer to linearity (Figure 4) and are formed preferentially when strong electron withdrawing residues are covalently bonded to selenium (*e*. *g*., **1** **j**). Intramolecular ChBs are formed wherever a donor of electron density is conveniently positioned (*e*. *g*., **1** **a**,**g**,**h**) and while these contacts tend to be remarkably short, they may also be poorly linear, both characteristics being a consequence of the geometric constrains inherent to the intramolecular arrangement. The presence of these contacts reshapes the MEP of the compound and prevents the finding of the σ‐hole corresponding to the contact (Table 1).

**Table 2:** Geometric parameters (distances and angles) of ChBs found in selected sulfinic acid derivatives from the CDS. V_S,max_ features of the same compounds (resulting from the MEP at 0.001 a.u. isosurface) are also reported along with V_S,max_ angular position.

Compound (Refcode)	S⋅⋅⋅Nu separation (pm), respective Nc [O/C−S⋅⋅⋅O angle (°)]				V_S,max_ at S (kJ/mol) [O/C−S⋅⋅⋅V_S,max_ angle (°)]		
	O=S⋅⋅⋅Nu	O−S⋅⋅⋅Nu	C−S⋅⋅⋅Nu		O=S⋅⋅⋅ V_S,max_	O−S⋅⋅⋅ V_S,max_	C‐S⋅⋅⋅ V_S,max_
**2** **b** ^[45]^ (MSUFAC)	^[a]^	^[a]^	^[a]^		87.1 [*169*]	79.9 [*145*]	^[a]^
**2** **c** ^[39]^ (TIZPEX)	^[a]^	^[a]^	328.5, 0.99 [175.78]		913.5^[d]^ [*169*]	891.2^[d]^ [*149*]	807.6^[d]^ [*170*]
**2** **d** ^[74]^ (EVIPIJ)	326.3, 0.98 [177.77]	^[a]^	318.3, [159.43]		^[b]^	^[c]^	8.8 [*160*]
**2** **e** ^[75]^ (KUGDEV)	^[a]^	326.0, 0.98 [159.66]	^[a]^		121.4 [*173*]	147.0 [154]	33.1 [*162*]
**2** **f** ^[76]^ (ZIRRAW)	^[a]^	^[a]^	305.2, 0.92 [172.49]		^[b]^	92.5 [*159*]	16.3 [*162*]
**2** **g** ^[77]^ (ASOROP)	312.4, 0.94 [166.37]	301.4, 0.91 [153.19]	^[a]^		147.6 [*172*]	185.5 [*141*]	102.2 [*151*]
**2** **h** ^[78]^ (RISROD)	^[a]^	277.9, 0.85 [157.71]	273.3, 0.84 [175.27]		1053.0 ^[d]^ [*169*]	1042.1 ^[d]^ [*165*]	1065^[d]^ [*161*]

[a] No ChB on the approximate elongation of the considered covalent bond. [b] No V_S,max_ on the approximate elongation of the considered covalent bond. [c] No V_S,max_ is detected probably due to the presence of the intramolecular O−S⋅⋅⋅Cl ChB. [d] The large and positive V_S,max_ values are related to the cationic nature of the molecule.

In order to support crystallographic information, surface MEPs of seleninic acid derivatives **1** **a**–**j** were computed (Gaussian16,^[63]^ B3LYP functional,^[64]^ cc‐pVTZ[^65^, ^66^] and ECP10MDF/cc‐pVTZ‐PP basis sets^[67]^). The 0.001 a.u. isovalue is used here as, according to Bader's recommendation,^[68]^ the surface that it generates contains 99% of the total electron density and it is a convenient estimation of the van der Waals surface of a molecule. The MEP on this isosurface of selenocystinic acid **1** **a** showed no position of local maximum electrostatic potential (σ‐hole) around selenium. A σ‐hole, while mainly associated with the depletion of electron density opposite to a covalent bond, is also a consequence of the whole molecular structure because the electrostatic potential at any point of a molecule results from the cumulative effect of the contributions of all the nuclei and electrons of the molecule, the greater contribution coming from the closer ones.^[69]^ σ‐Holes have not been found around selenium in **1** **a** probably due to the close proximity of the carboxylate anion which is chalcogen bonded to selenium on the elongation of the HO–Se covalent bond. No σ‐hole was found also on the elongation of the O–Se covalent bond in **1** **g**,**h** where an intramolecular O−Se**⋅⋅⋅**O ChB is found in corresponding crystals and forms a non‐covalent five membered ring in which the oxygen of the nitro groups plays the same role of the carboxylate oxygen in **1** **a**. It is now useful to comment on the dependence of the σ‐holes on the isovalue used to construct the MEP surface. As mentioned above, the 0.001 a.u. isovalue excellently estimates the van der Waals surface of a molecule and it is the benchmark for determining MEPs when interactions analysis is pursued. But the Se**⋅⋅⋅**O distances of ChBs observed in **1** **a** and other seleninic acid derivatives presented in Table 1 are shorter than the sum of the corresponding van der Waals radii (Nc <1). It is thus informative to consider also MEPs on smaller surfaces, *i*. *e*. to use increased isovalues. MEPs of selenocystinic acid (**1** **a**), methane and pentafluorobenzene seleninic acids (**1** **b** and **1** **d**), and the ester **1** **g** were calculated on the electron density surface of 0.06 a.u. As expected,^[70]^ MEP values are more positive on these smaller surfaces and three σ‐holes are found around selenium for all the four derivatives, namely σ‐holes appear also where missing on the 0.001 a.u. surface. Specifically, the V_S,max_ (kJ/mol) opposite to the O=Se, O−Se, and C−Se bonds are 1439, 1449, and 1423 for **1** **a**, 1534, 1548, and 1479 for **1** **b**, 1549, 1591, and 1593 for **1** **d**, and 1455, 1600, and 1544 for **1** **g**. These findings further support the rationalization of observed Se**⋅⋅⋅**O ChBs as σ‐hole interactions.

**Figure 5:**
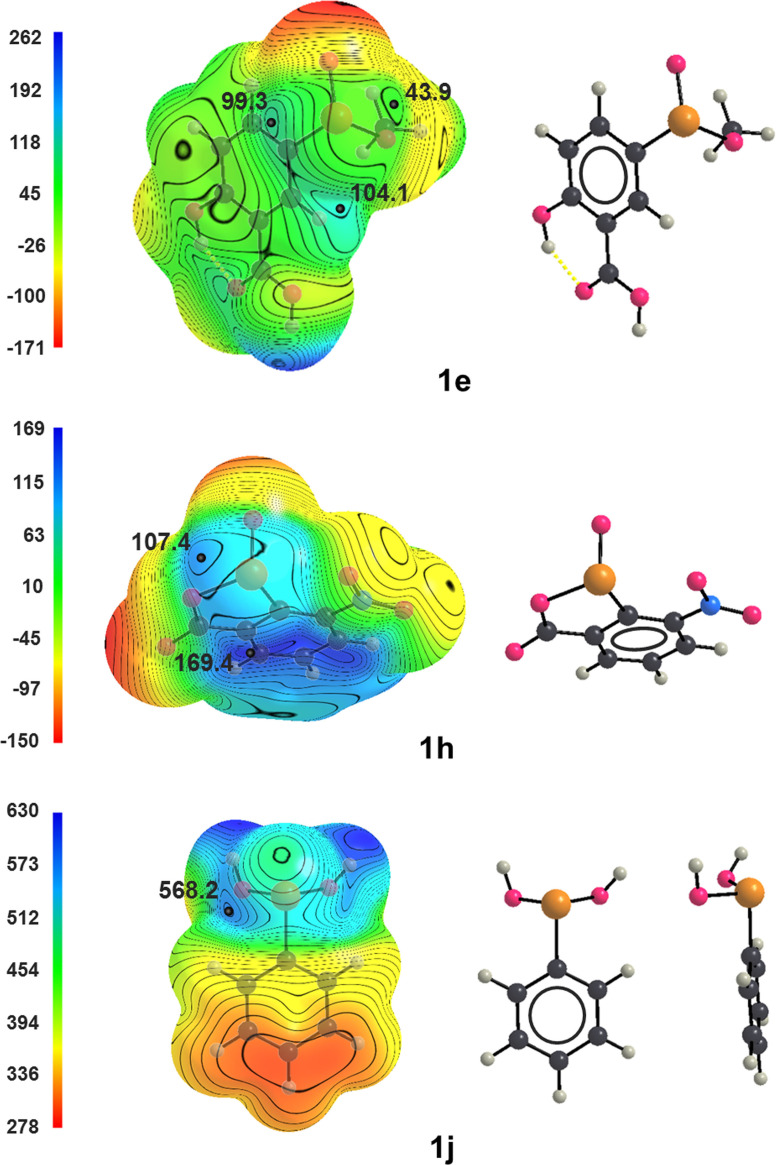
Computed electrostatic potential on the 0.001 a.u. molecular surfaces of seleninic acid derivatives **1** **e** (top), **1** **h** (middle), and **1** **j** (bottom). Black dots are σ‐holes; the electrostatic potential is in kJ/mol. Color codes in structural formulas: gray, carbon; whitish, hydrogen; red, oxygen; blue, nitrogen; ocher, selenium.

QTAIM Analysis afforded computational confirmations that short contacts observed in **1** **a** crystal are not crystal packing effects. In fact, the three bond critical points (BCPs) related to the three Se**⋅⋅⋅**O short contacts observed in **1** **a** crystal are found and these BCPs are consistent with the calculated MEP, since they are localized within the 0.001 a.u. surface. Interestingly, the electron density values at the three BCPs parallel the length of the respective ChBs. The BCPs with the highest and lowest electron densities (*i*. *e*., 0.036 and 0.010 a.u.) are along the paths of the intramolecular HO−Se**⋅⋅⋅**O and the intermolecular O=Se**⋅⋅⋅**O contacts, namely the shortest and longest ChBs. The intermediate value of electron density (0.15 a.u.) is associated with the BCPs of the medium length ChB, i. e., the C−Se**⋅⋅⋅**O interaction. σ‐Holes were frequently found around selenium in the seleninic acid derivatives **1** **b**–**j** and corresponding V_S,max_ values are reported in Table 1. These values are typically quite positive. For instance, in neutral and cationic seleninic acid derivatives (*e*. *g*., **1** **b**,**h** and **1** **j**, respectively) they can be as positive as V_S,max_ values in neutral and cationic diselenide derivatives.^[71]^ Diselenenides are robust ChB donors[^71^, ^72^, ^73^] and this confirms crystallographic indications afforded by **1** **a** that seleninic acid derivatives can act as robust ChB donors. **1** **j** Is a cationic species and the electrostatic potential is positive in the whole molecule (Figure 5, bottom), being more positive close to the protonation site. A σ‐hole is present on the elongation of only one of the two O−H groups, probably as a consequence of the different orientation of the protons in the two cases.

**Figure 6:**
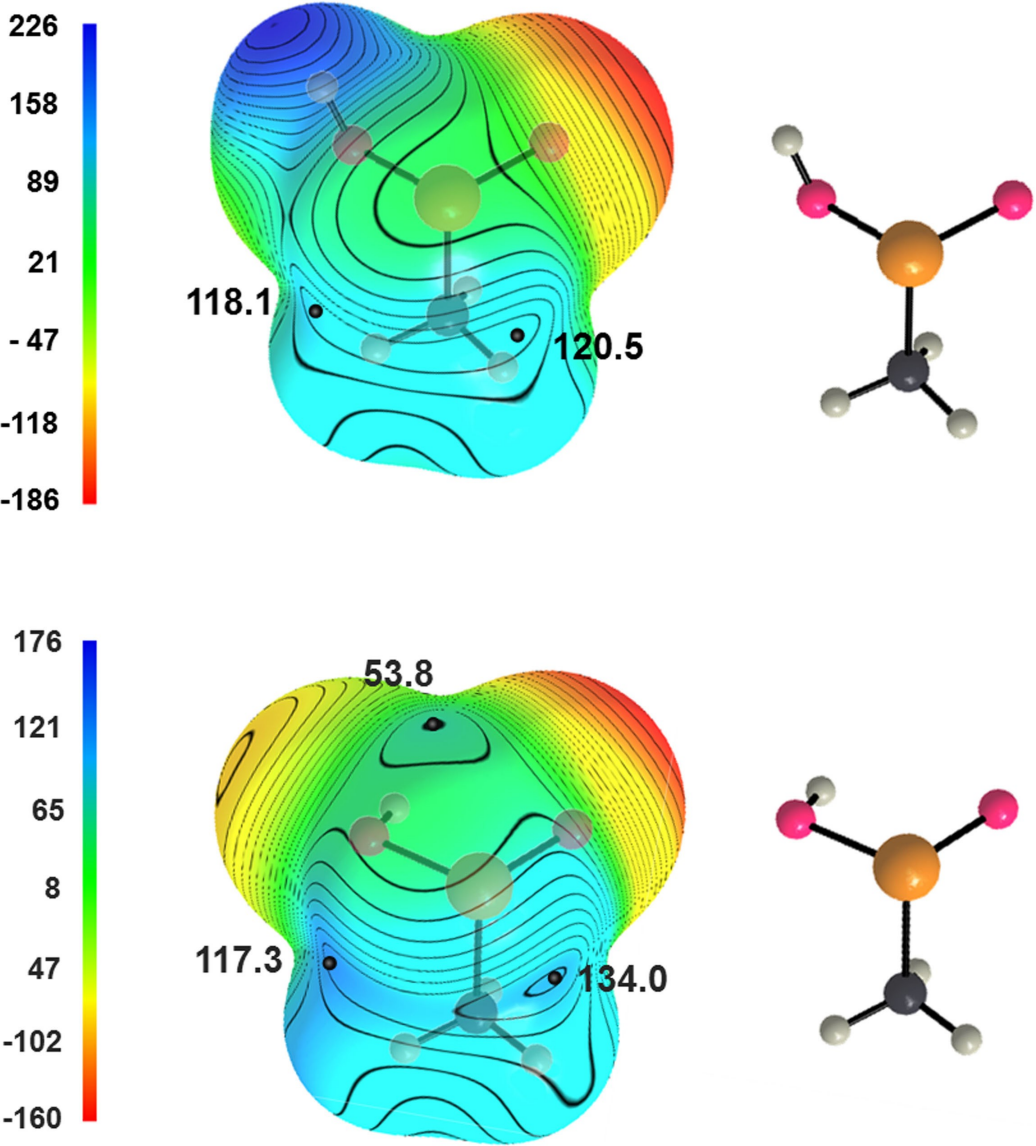
Computed electrostatic potential on the 0.001 a.u. molecular surfaces of methaneseleninic acid (**1** **b**) when adopting the conformation in the crystal^[44]^ (top) and in the optimized structure of the isolated molecule (bottom). Black dots are σ‐holes; the electrostatic potential is in kJ/mol. Color codes in structural formulas: gray, carbon; whitish, hydrogen; red, oxygen; ocher, selenium.

The impact of the orientation of O−H proton on the σ‐holes is observed also in methaneseleninic acid **1** **b**. In the conformation adopted in the solid two σ‐holes are found (Figure 6, top) and they are opposite to HO−Se and O=Se covalent bonds, namely at the sites where ChBs are observed in the crystal. In the most stable conformation computed for the isolated molecule the O−H group adopts a different arrangement and the molecule shows the presence of three σ‐holes (Figure 6, bottom); the one opposite to the C−Se bond is the least positive and in the crystal the C−Se**⋅⋅⋅**O contact is slightly above the sum of O and Se van der Waals radii (349 *vs*. 342 pm). The QTAIM analysis of **1** **b** confirms the attractive nature of the short contacts observed in its crystal. Three bond paths are found connecting three different oxygen atoms to the selenium atom. The electron density at the BCPs is 0.010, 0.008, and 0.005 a.u. for the HO–Se**⋅⋅⋅**O, O=Se**⋅⋅⋅**O, and C−Se**⋅⋅⋅**O ChBs, respectively, and these relative densities match with the relative lengths of corresponding interactions (315.6, 331.3, and 348.8 pm).

Both crystallographic O/C−Se**⋅⋅⋅**O angles and computed O/C−Se**⋅⋅⋅**V_S,max_ angles show in some cases non minor deviations from linearity (Table 1). This is another consequence of the relevance of the whole molecular structure on computed σ‐holes and ChBs involving them. A major cause of this partial disarray of the position of the V_S,max_ and of the ChBs involving them is probably the presence of the lone pair on selenium. Three covalent bonds and a lone pair are present on selenium in seleninic acid derivatives and on pnictogen atoms in their trivalent derivatives and these latter compounds show a deviation of experimental substituent‐Pn**⋅⋅⋅**nucleophile angles and of computed substituent‐Pn**⋅⋅⋅**V_S,max_ angles (Pn=P, As, Sb, Bi) typically greater than in halogen and tetrel atoms derivatives, wherein the disturbing single lone pair is missing.^[35]^

Analyses of sulfinic acid derivatives reported in the CSD, calculation of their MEPs, and QTAIM analyses proved that also the sulfur atom of these systems can function as ChBs donor.

None of sulfinic acid derivatives found in the CSD shows the presence of three ChBs, approximately two per cent and twenty per cent of these compounds show the presence of two and one ChB, respectively (Table 4S) and this reveals that the sulfinic acid functionality is less prone than the seleninic acid one to act as ChB donor. This supports the rationalization of the considered interactions as ChBs. In fact, it is well established that on increasing the molecular weight of an element of the 14, 15, 16, or 17 group of the periodic table (namely on increasing the polarizability of the element and decreasing its electronegativity) the respective tendency to form a tetrel,^[78]^ pnictogen,^[80]^ chalcogen,^[33]^ or halogen^[62]^ bond increases.

**Scheme 4:**
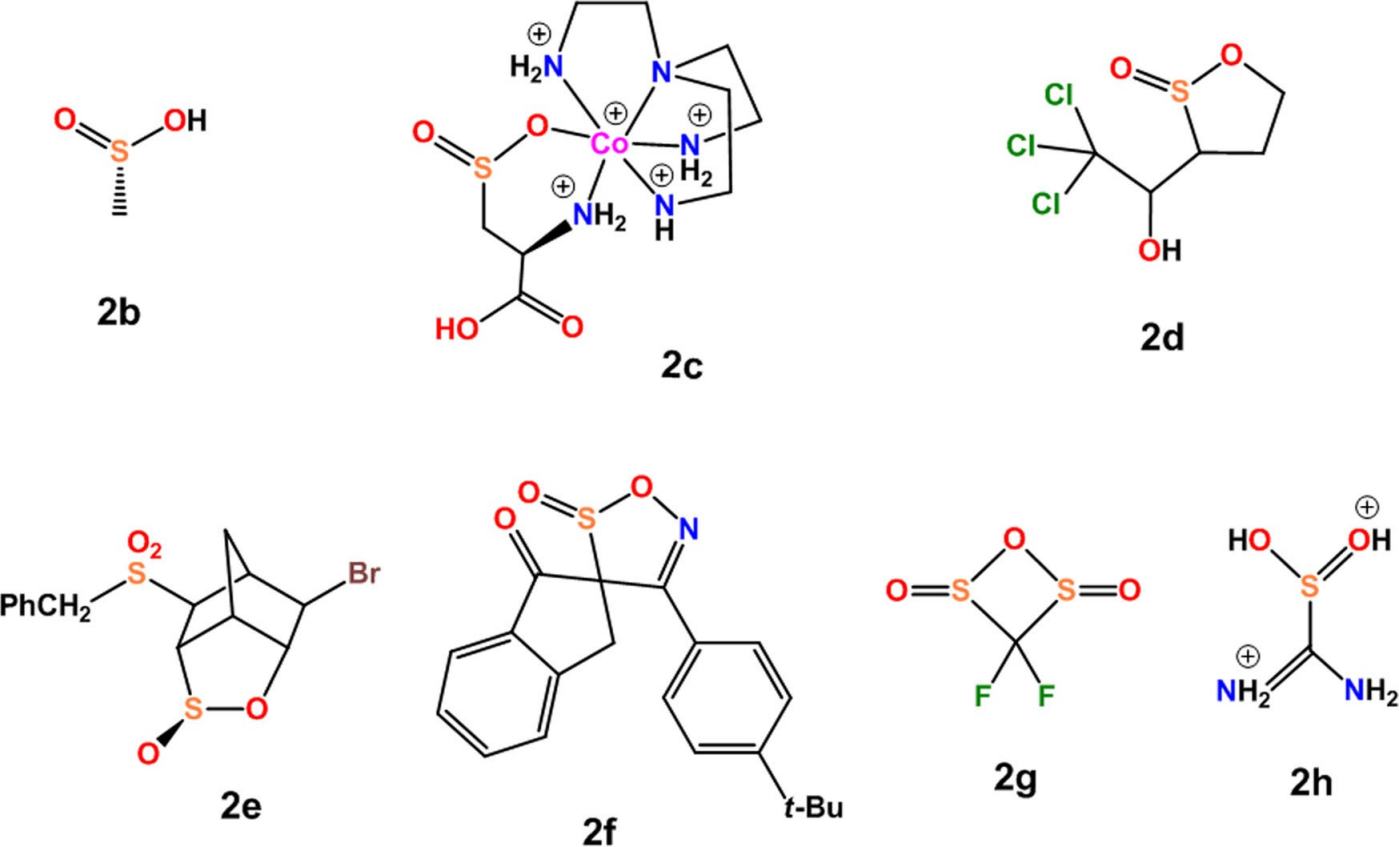
Chemical formulas of selected sulfinic acid derivatives from the CSD showing the presence of ChB(s) in respective crystals.

**Figure 7:**
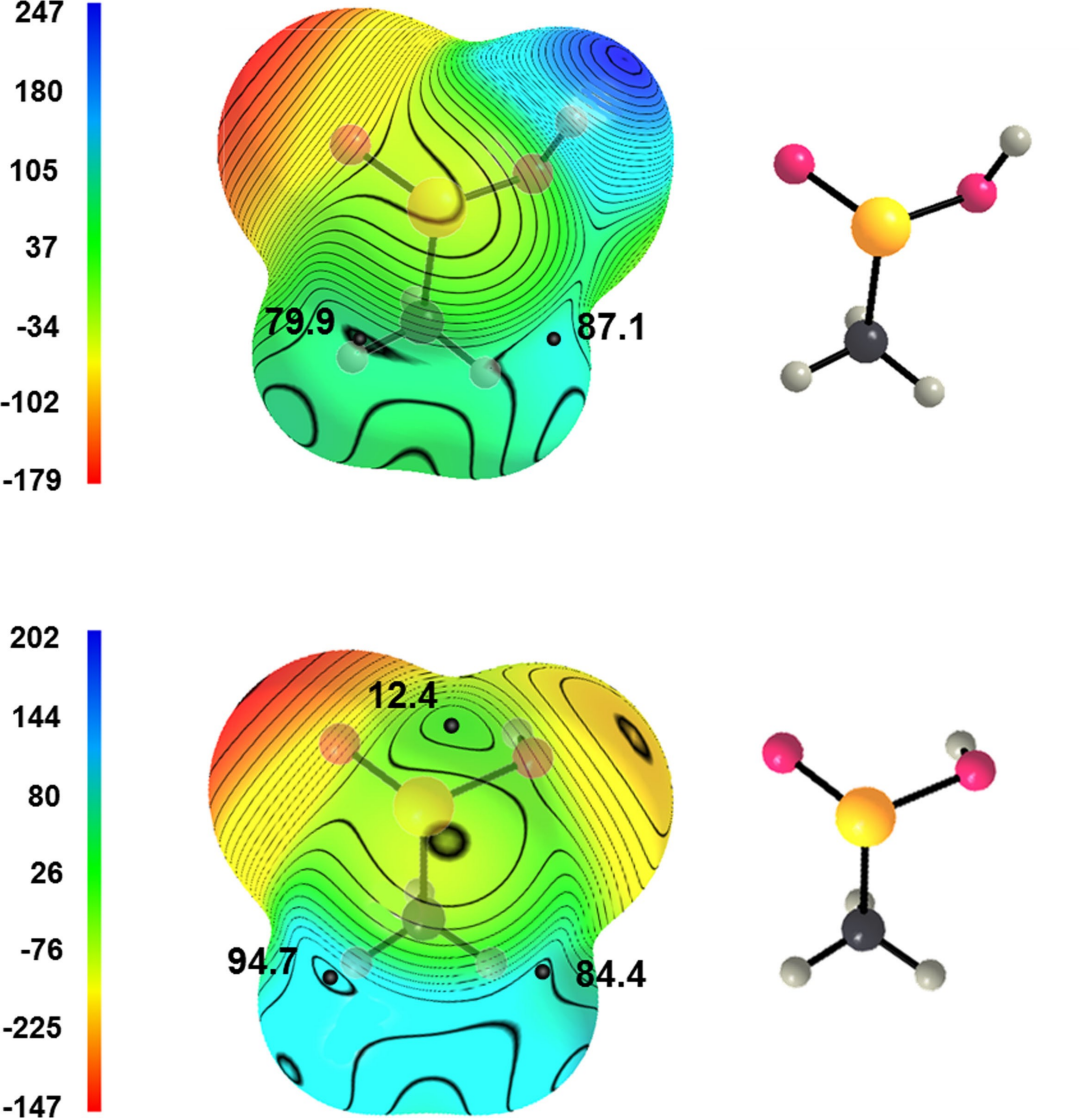
Computed electrostatic potential on the 0.001 a.u. molecular surfaces of methanesulfinic acid (**2** **b**) when adopting the conformation in the crystal^[45]^ (top) and in the optimized structure of the isolated molecule (bottom). Black dots are σ‐holes; the electrostatic potential is in kJ/mol. Color codes in structural formulas: gray, carbon; whitish, hydrogen; red, oxygen; yellow, sulfur.

Attention was focused on compounds **2** **b**–**h** (Scheme 4) wherein ChB(s) are present. Selected geometric parameters from respective crystals and pertinent calculated features are reported in Table 2. Some general features of ChBs formed by sulfinic acid derivatives clearly parallel those of seleninic analogues. For instance, the interaction is afforded by different functional groups containing the C−S(=O)O− moiety, the electron donor site can be both an anion and a neutral lone pair possessing atom, shorter contacts tend to be closer to linearity (Figure 1S) and are formed preferentially when strong electron withdrawing residues are covalently bonded to sulfur (e. g., **2** **f**–**h**, Scheme 4). Methanesulfinic acid **2** **b** shows the presence of two similar σ‐holes opposite to the HO−S and O=S bonds in the conformation adopted in the crystal^[45]^ and three σ‐holes in the most stable conformation computed for the isolated molecule, the σ‐hole opposite to the C−S bond being the least positive (Figure 7). Similar to methaneseleninic acid **1** **b**, this is probably a consequence of the different position of the O−H group in the two adopted conformations. QTAIM analysis of **2** **b** returns bond paths for HO−S**⋅⋅⋅**O and C−S**⋅⋅⋅**O interactions and corresponding electron density values at BCP are quite small (0.006 and 0.005 a.u.), consistent with crystallographic separations (337.4 and 344.0 pm, respectively) which are above the sum of S and O van der Waals radii (332 pm).

## Conclusions

The crystal structure of L‐selenocysteine seleninic acid (**1** **a**), obtained through controlled oxidation of L‐selenocystine, is reported. In the solid the amino acid is present as a carboxylate zwitterion and the selenium atom forms three ChBs which heavily affect the conformation of the compound as well as the overall packing of the crystal. Notably, the HO−Se**⋅⋅⋅**O ChB has a Nc value as small as 0.69. A survey of seleninic acid derivatives in the CSD confirms that the tendency of the C−Se(=O)O− functionality to act as a ChB donor is robust enough to systematically influence the interactional landscape in the solid. QTAIM analysis proves the attractive nature of the short contacts observed in crystals. Calculation of MEP reveals that remarkably positive σ‐holes can frequently be found opposite to the covalent bonds at selenium. The offset of these σ‐holes from the elongation of the covalent bonds responsible for their presence can be non‐minor suggesting that the poor linearity of some of the ChBs found in crystals is an inherent feature of these interactions rather than a crystal packing effect. CSD searches and modelling (QTAIM analyses and MEP calculations) reveal that also the sulfinic acid moiety can function as a ChB donor. As expected, sulfur in sulfinic acid derivatives is less prone to act as electrophile than selenium in seleninic acid derivatives. Selenocysteine seleninic acid (**1** **a**) and cysteine sulfinic acid (**2** **a**) are present in the active site of enzymes involved in controlling oxidative stress and are actively involved in the ROS deactivation catalytic cycle. The recognition of the electrophilic behavior of selenium and sulfur in **1** **a** and **2** **a** allows for a better understanding at the atomic level of the mechanism of action of the enzymes containing them. This is quite important as the relevance of the electrophilic behavior of sulfur and selenium atoms in GPx mimics containing these elements has been recognized.^[29]^

Finally, thanks to the results presented in this paper, the C−Se(=O)O− and C−S(=O)O− functionalities enter the toolbox of the supramolecular chemists as two new and effective moieties for controlling molecular conformation and packing *via* ChB formation. Notably, the ChB donor ability of the former functionality may equal that of other robust selenium containing moieties.[^72^, ^73^]

## Experimental Section

### Materials and General Procedures.

^1^H (400 MHz), ^13^C (100 MHz) and ^77^Se NMR (95 MHz) were recorded at Bruker AV 400 MHz and Bruker AV 500 MHz spectrometers at 25 °C. Chemical shifts were referenced to TMS (^1^H, ^13^C) as internal and Me_2_Se (^77^Se) as external standards. Electron spray mass spectra (ESI‐MS) were recorded on an Agilent 6220 TOF MS system (Santa Clara, CA, USA) with a multimode ESI/APCI source in positive mode. IR spectra were recorded using an Agilent Technologies Cary 630 ATR‐IR instrument within the range 4000–700 cm^−1^. Melting points were recorded in capillary tubes on a Veego VMP‐1 instrument and are uncalibrated. The elemental analyses were performed on a Thermo Finnigan FLASH EA 1112 series CHN analyzer along with a thermal conductivity detector (TLD).

### Synthesis of L‐ selenocysteine seleninic acid (1 a).

A 4 : 1 mixture of methanol and water (10 mL) was added into a 15 mL vial containing L‐selenocystine (0.10 g, 0.30 mmol) and the resulting solution sonicated for one minute. Then, 4 equivalents of hydrogen peroxide (28 μL, 1.2 mmol) were added dropwise into the vial over a period of 10 minutes and the reaction vessel was kept overnight at 10 °C. Colorless block crystals of selenocystinic acid **1** **a** were obtained, washed with methanol and vacuum dried for further analyses. Yield: 0.07 g (60%); m.p. 210–212 °C (dec.); ^1^H NMR (400 MHz; D_2_O+NaOD): *δ*=5.98 (t, *J*=5.67, 6.05 Hz, 1H), 5.75(dd, *J*=4.59, 4.59 Hz, 1H), 5.61 (dd, *J*=7.39, 7.65 Hz, 1H) ppm. It should be noted that 4H were not observed owing to H/D exchange; ^13^C NMR (400 MHz; D_2_O+NaOD): *δ*=38.13, 58.72 and 183.39 ppm; ^77^Se NMR (95 MHz; CD_3_OD): *δ*=1221 ppm; ESI‐MS (*m*/*z*) calculated for C_3_H_7_NO_4_Se: 200.9540 [M]^+^; found: 200.8365. Elemental analysis calculated for C_3_H_7_NO_4_Se (%): C, 18.01; H, 3.53; N, 7.00; found: C, 18.67; H, 3.06; N, 6.01.

### X‐Ray Crystallography

The single crystal X‐ray diffraction data for L‐selenocysteine seleninic acid (**1** **a**) were measured on a Rigaku Synergy‐S diffractometer microsource Cu−Kα radiation (λ=1.54184 Å) at 123 K. Data processing was conducted using CrysAlisPro software suite.^[81]^ The structure was solved using direct methods with SHELXT^[82]^ and refined using full‐matrix least‐squares methods against F^2^ with SHELXL‐2018,^[83]^ in conjunction with the Olex2^[84]^ graphical user interface. All hydrogen atoms were placed in calculated positions using the riding model. **Crystal Data** for C_3_H_9_NO_5_Se (*M* =218.07 g/mol): orthorhombic, space group P2_1_2_1_2_1_ (no. 19), *a*=5.93050(10) Å, *b*=10.72050(10) Å, *c*=11.06300(10) Å, *V*=703.363(15) Å^3^, *Z*=4, *T*=122.99(10) K, μ(CuKα)=7.091 mm^−1^, *Dcalc*=2.059 g/cm^3^, 4056 reflections measured (11.494°≤2θ≤151.5°), 1318 unique (*R*_int_=0.0183, R_sigma_=0.0173) which were used in all calculations. The final *R*_1_ was 0.0168 (I>2σ(I)) and *wR*_2_ was 0.0445 (all data).

The data can be obtained free of charge from The Cambridge Crystallographic Data Centre via www.ccdc.cam.ac.uk/data_request/cif. (CCDC number: 2044358)

### Computational Aspects

All calculations presented were performed on Gaussian16^[63]^ using the B3LYP functional^[64]^ with cc‐pVTZ basis sets.[^65^, ^66^] A small‐core fully relativistic effective core potential was employed on selenium (ECP10MDF) along with the corresponding cc‐pVTZ‐PP basis.^[67]^ The geometric coordinates extracted from experimental crystal structures were used for non‐hydrogen atoms; length and orientation of C−H bonds were optimized without constraints while length only of X−H bonds (X≠C) was optimized (orientation was maintained unchanged).

## Conflict of interest

The authors declare no conflict of interest.

## Supporting information

As a service to our authors and readers, this journal provides supporting information supplied by the authors. Such materials are peer reviewed and may be re‐organized for online delivery, but are not copy‐edited or typeset. Technical support issues arising from supporting information (other than missing files) should be addressed to the authors.

Supporting Information
