# Supplementary material for: Chalcogen Bonds in Selenocysteine Seleninic Acid, a Functional GPx Constituent, and in Other Seleninic or Sulfinic Acid Derivatives
Source: Chem Asian J. 2021 Jul 16;16(16):2351–60. doi: 10.1002/asia.202100545 (PMC8456948; doi:10.1002/asia.202100545)
Supplement: Supplementary file 1 — Supporting Information [file ASIA-16-2351-s001.pdf]

CHEMISTRY
AN ASIAN JOURNAL

Supporting Information

Chalcogen Bonds in Selenocysteine Seleninic Acid, a Functional GPx Constituent, and in Other Seleninic or Sulfinic Acid Derivatives

Abhishek Tripathi, Andrea Daolio, Andrea Pizzi, Zhifang Guo, David R. Turner, Alberto Baggioli, Antonino Famulari, Glen B. Deacon,* Giuseppe Resnati,* and Harkesh B. Singh*© 2021 The Authors. Chemistry - An Asian Journal published by Wiley-VCH GmbH. This is an open access article under the terms of the Creative Commons Attribution License, which permits use, distribution and reproduction in any medium, provided the original work is properly cited.

page

1. Cambridge Structural Database searches…………………………….3

2. MEP studies………………………………………………………………7

3. Characterization of 1a…………………………………………………...9

4. Crystallographic Details……………………………………………..11

5. References……………………………………………………….......15

1. Cambridge Structural Database searches.

Table 1S. Selected geometrical parameters (separations in pm, angles in degrees) of **1a** and prototype sulfinic, seleninic, and tellurinic acids (Ar/R–ChO_2H, Ch=S, Se, Te).

Compound[a]	Ch–O(1)	Ch–O(2)	Ch–C(1)	O(1)–Ch–O(2)	O(1)–Ch–C(1)	O(2)–Ch–C(1)
**1a**	178.6(2)	167.8(2)	195.3(3)	101.40(12)	89.68(12)	102.26(13)
**$MeSeO_2H$[1]**	175.3(2)	166.9(2)	191.4(4)	103.50(12)	93.59(15)	100.79(15)
**$MeSO_2H$[2]**	159.5(7)	149.5(7)	178.1(10)	107.50(7)	95.00(1)	104.00(1)
**$ArSeO_2H$[3]**	175.2(4)	165.8(3)	195.7(4)	98.20(2)	98.20(2)	102.90(2)
**$PyTeO_2H$[4]**	196.6(2)	183.4(2)	212.8(3)	96.00(1)	92.80(1)	103.00(1)

[a] Ar=C_6H_4-2-$CONHC_6H_5$; Py=C_6H_4-2-(2'-C_5H_4N).

Table 2S. List of Refcodes of structures obtained from a CSD survey for the –C–Se(O)O– moiety wherein selenium is trivalent, the carbon/selenium bond is "single", and the two oxygen/selenium bonds are "any". A ChB is identified when there is an intra- or intermolecular C/O–Se···Nu contact wherein Nu=N, P, O, S, Se, F, Cl, Br, I, the Se···Nu separation is below the sum of Se and Nu van der Waals radii, and the C/O–Se···Nu angle is > 155°. Refcodes for structures displaying one, two, or three ChBs are in blue, red, or green, respectively (in black Refcodes of structures showing no ChB).

COXMEK	BOVGOI	KAJNUH	POHZET	TARKUU	XEHWAK
COXMIO	CEYTUX	KUTRAS	POHZIX	TARLAB	YABSAX
COXMOU	CEYVAF	LAHZOK	POHZOD	TARLEF	YABSEB
COXMUA	CLBSEA	LAHZUQ	QOLHAC	TARLIJ	YABSIF
COXQUE	COKLIY	LAYFAT	QOZFOC	TARREJ	YABSOL
FUBWEH	DAMTET	LOBSAZ	QOZFUI	TESEAN	YABSUR
FUBWIL	DIPKIZ	MANMOF	QOZTIK	TOMVAT	YABTAY
JUCRUX	DIPKOF	MANMUL	RULREW	TUNBUB	YACDAJ
XUHNOG	DIPKUL	MANNAS	RULRIA	TUNMOG	YACDEN
ADEFEX	DIPLAS	MANNEW	SEWMIT	TUNMUM	YACDIR
ADEFIB	GEKWIC	MSNSEL	SEWRIY	TUNNAT	YANPUA
ADEFOH	IBUQUR	NAXBEU	SEWSEV	TUNNEX	YANQAH
ADEFUN	IGIKOA	OBIZOQ	SEWSEV01	WIKYAT	YANQEL
AESELA	IJORIJ	OKUCII	SOXROM	WIKYEX	YODBAX
AWAVOJ	ISUJEM	PAFQOF	SUMCAF	XEHHIB	ZZZLUU
BATFAD	JELREA	PAFQUL	SUMCEJ	XEHHOH	ZZZLWC

BEMNOY	KAJNIV	PAFTEY	SUMCIN	XEHVOX	GUCLEY
BENSEA	KAJNOB	POHZAP	TARKOO	XEHVUD	

Table 3S. Refcodes of structures in the CSD wherein the –C–Se(O)O– moiety shows a O–Se···Nucleophile, O=Se···Nucleophile, or C–Se···Nucleophile ChB. See caption of Table 2S for criteria used to identify the –C–Se(O)O– moiety and the ChBs.

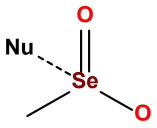			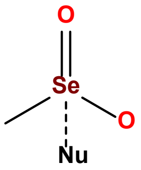			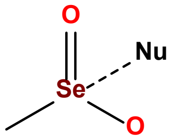		
**Refcode**	**O–Se···Nu Angle**	**Se···Nu Distance**	**Refcode**	**O–Se···Nu Angle**	**Se···Nu Distance**	**Refcode**	**O–Se···Nu Angle**	**Se···Nu Distance**
JUCRUX	166.697	2.589	JUCRUX	161.928	2.897	BATFAD	174.705	2.923
AESELA	178.31	3.458	AESELA	167.236	3.264	BENSEA	177.948	3.165
AWAVOJ	175.425	3.156	AWAVOJ	174.262	3.313	CLBSEA	177.323	3.153
BATFAD	157.778	3.027	BENSEA	169.644	3.328	DIPKIZ	159.571	3.219
BOVGOI	176.033	3.111	DIPKIZ	158.751	3.414	DIPKOF	157.994	3.069
CLBSEA	167.846	3.321	DIPKOF	155.376	3.326	DIPKUL	159.116	3.089
DIPKIZ	168.886	3.327	DIPKUL	155.739	3.321	DIPLAS	159.283	3.069
DIPKOF	168.858	3.309	DIPLAS	155.437	3.292	GEKWIC	161.571	2.95
DIPKUL	170.078	3.299	GEKWIC	173.451	2.781	ISUJEM	169.845	3.13
DIPLAS	170.569	3.325	ISUJEM	160.453	3.262	NAXBEU	173.293	2.912
LAYFAT	162.839	2.76	LAYFAT	177.119	3.294	QOLHAC	176.778	2.907
NAXBEU	157.282	3.059	PAFQUL	170.569	2.739	SUMCEJ	176.748	3.048
OKUCII	164.259	2.994	PAFQUL	170.793	2.827	SUMCIN	167.853	3.182
QOLHAC	163.352	2.749	RULRIA	167.952	3.011			
RULREW	167.823	2.472	TESEAN	157.691	3.108			
RULRIA	165.941	2.851						
SUMCIN	177.505	2.946						
TESEAN	159.246	2.701						
TOMVAT	168.197	2.46						

Table 4S. List of Refcodes of structures obtained from a CSD survey for the –C–S(O)O– moiety wherein sulfur is trivalent, the carbon/sulfur bond is "single", and the two oxygen/sulfur bonds are "any". ChB are identified when there is an intra- or intermolecular C/O–S···Nu contact wherein Nu=N, P, O, S, Se, F, Cl, Br, I, the S···Nu separation is below the sum of S and Nu van der Waals radii, and the C/O–S···Nu angle is > 155°. Refcodes for structures displaying one or two ChBs are in blue or red, respectively (in black Refcodes of structures showing no ChB).

BOLPEA	DARWUP	JEKVIF	OWUJUN	SUWGIA	YAPWET
BOLPEA01	DAVBAF	KACYUI	PATVAH	TAFZAB	YEJKAB
KUBCAO	DEFMOQ	KAGNOW	PELZEL	TAKFOA	YETBEH
KUBCAO01	DUCBAF	KAGNOW01	PELZIP	TEFVOP	YETJIR
MOWXAA	DUCDAG	KAHPOY	PELZOV	TICPUT	YIXPAW
QOXQUS	EKUMEC	KAHVIB	PEPSNI10	TIKBIZ	YIXPAW10
QOXSII	ETUBEB	KAJYUS	PITVEV	TIYKUK	YULXIM
ADURUP	EVIPIJ	KAJZAZ	PITVIZ	TIZPEX	ZADXOU
AJOREX	EWOVIW	KECCIG	PODFET	TMSNMS	ZADXUA
AJORIB	FALRAL	KOVCIH	PORBUW	TMSNMS01	ZADYAH
AJOROH	FALREP	KOVCIH01	PORCAD	TOLLUB	ZADYEL
ASOROP	FALREP01	KOXCIJ	PORCEH	TPOXTO	ZAMCUO
AWIMOK02	FALREP02	KUGDEV	PORCIL	TSLFCU	ZAMDAV
BACZIO	FALSOA	LAQKOD	PURCOU	TUHHIN	ZAWHUC
BAJREJ	FAYYAG	LEJQID	PYSLCU	UDOYET	ZAWJAK
BAPNEL	FEBUTS10	LIJLEY	QEBXOK	UFEHEU	ZAWJEO
BECSOR	FIMGUD	LIPLII	QEBXUQ	UGODIF	ZAWJIS
BECSOR01	FIWCEU	MAWGUN	QIFNIC	ULUTEA	ZEDJAT
BEFHIG	FIWCIY	MAZWAL	QIKWOZ	VAJTUX	ZEKBOG
BEJBEA	FOCGIN	MCDXBT	QORFUA	VALWOU	ZESKAM
BEXSOM	GAQHIP	MEMXAE	QOTBIK	VAYTEV	ZIPBIL
BISLEX	GEJVOH	MIJWEK	RESKEF	VEVDUV	ZIRQOJ
BOGRIA	GELTUM	MILSOP	RIKPOQ	VEZPOG	ZIRQUP
BSTCFE	GESCAI	MNIBZS	RISROD	WACMIZ	ZIRRAW
BUNXEN	GEYBAN	MSUFAC	SACTMT10	WALBIV	ZIRRIE
CEQMOA	GIJLOB	MUKGOO	SALCAJ	WALBUH	ZOPQEC
CEQMUG	GUBMIB	MURVOM	SANXTO	WANXAN	ZUKNAU
CETWON	GUBMOH	NAHMSD	SANXTP	WANXIV	ZUWPIQ
CEVREB	GUBNAU	NAJDOR	SECSEY	WEMBUO	ZUYYOH
COFMOZ	GUETSA	NECMIS	SECSEY01	WOSVUV	ZZZJPG
COFMOZ01	GUGPUU	NEKTUT	SEHFIX	WUGBIJ	ZZZMSQ
COJFIS	HEASLF10	NIQFAX	SEHFOD	WULMAS	ZZZMVU
COJGAL	HEGXAS	NONTOA	SEHFUJ	WUZJIM	GUBMAU
COJGEP	HEGXAS10	OLICIV	SEMDIX	XEWRUN	GUBMUO

COKDEL	HUPZEX	OMOZAT	SEMDOD	XICQAC	GUBNEZ
CPFESO10	IBINIS	OMOZEX	SEYZEE	XICVOU	GUBNID
CUJSOP	IDOLUK	OPYMSU	SIKHEZ	XINXUN	GUBNOJ
CUYWIC	IMUVIW	OTETAK	SONVAS	XTBCOC	TUDQOB
DAFCOC	JECLAE	OVEQIQ	SUNPOG	YAPREN	TUDRAO

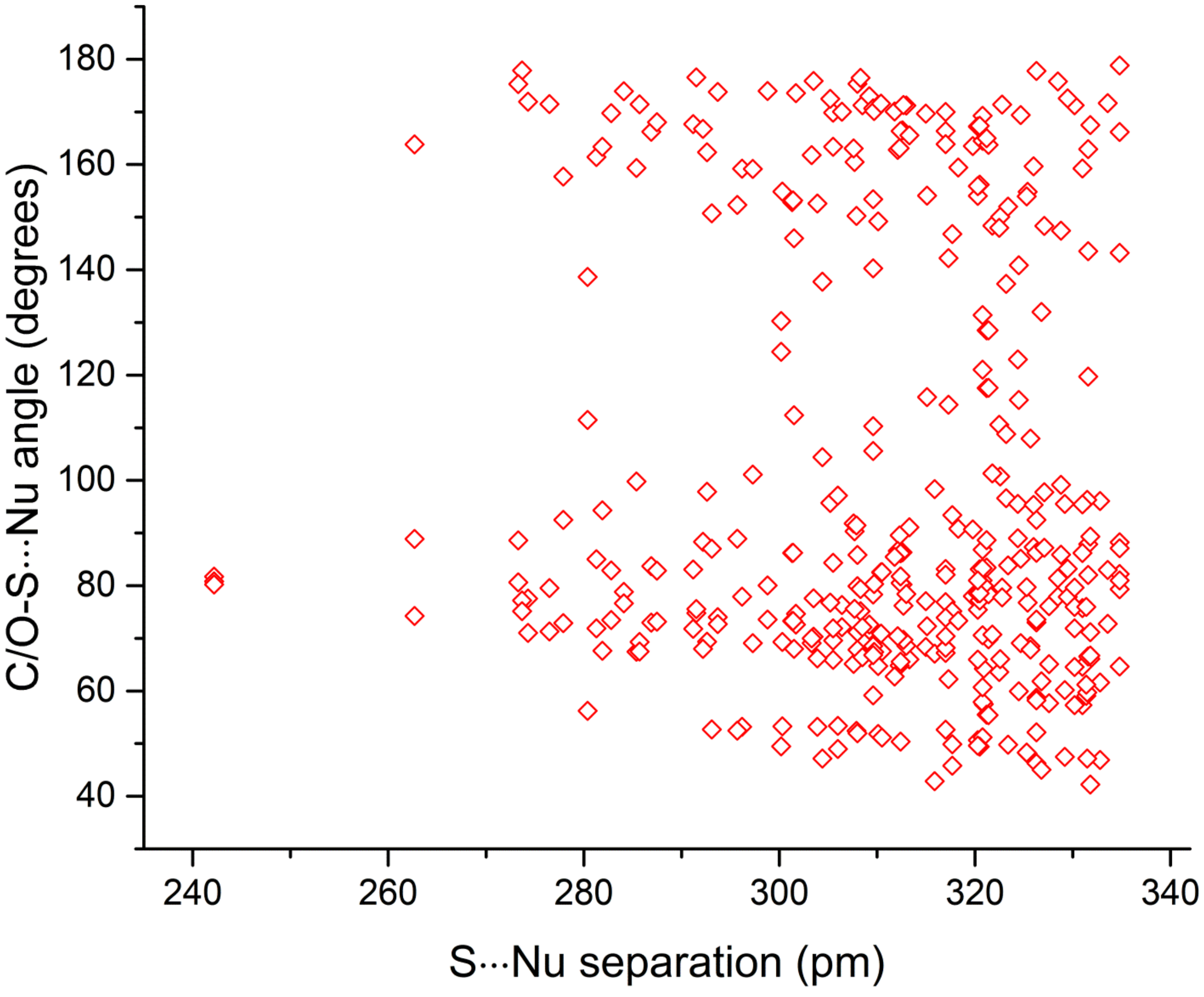

Figure 1S. Scatterplot of C/O–S···Nucleophile ChBs versus S···Nucleophile distances for structures present in the CSD. See caption of Table 4S for criteria used to identify the –C–S(O)O– moiety and the ChBs.

2. MEP studies.

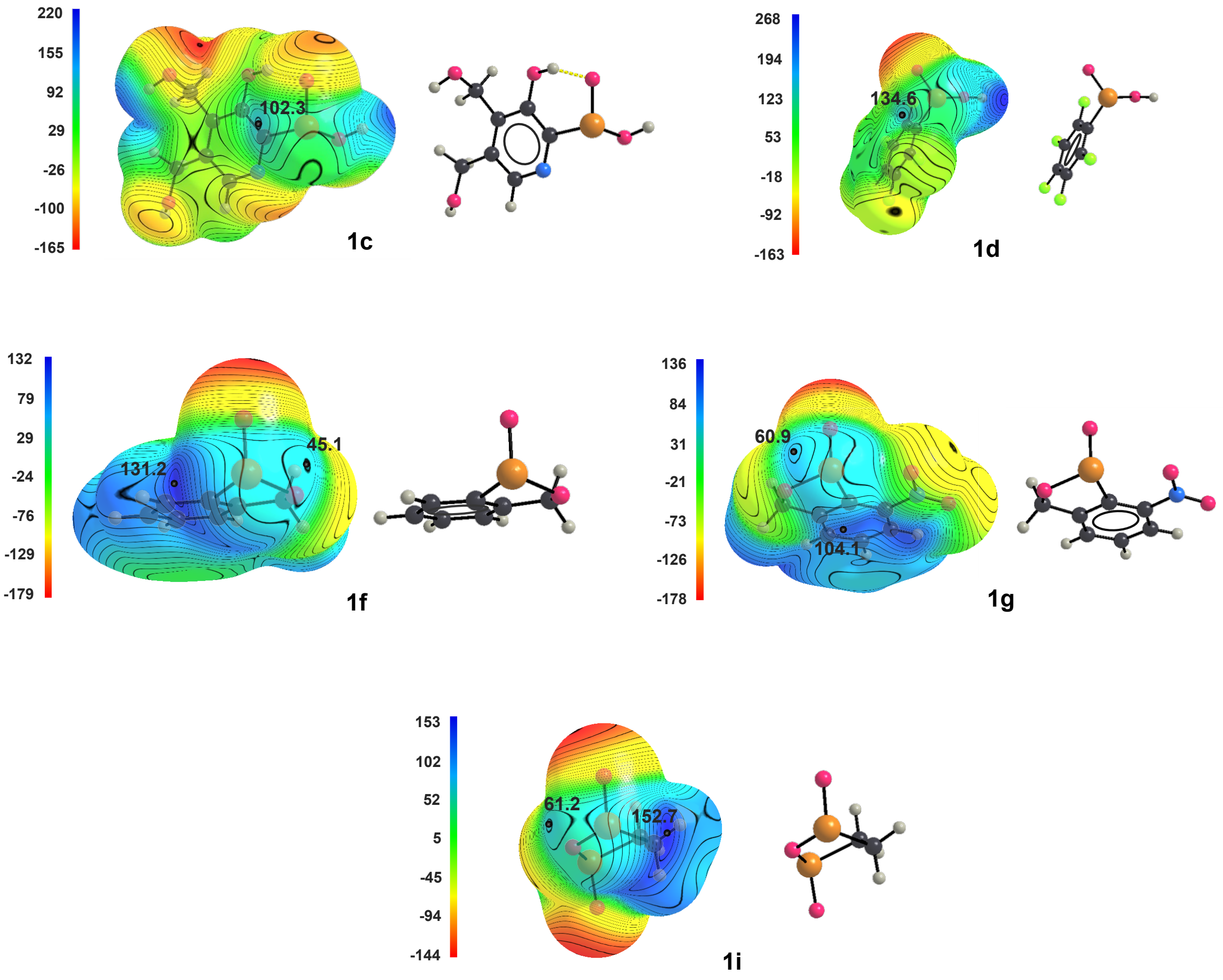

Figure 2S. Computed electrostatic potential on the 0.001 au molecular surfaces of seleninic acid derivatives **1c**, **d**, **f**, **g**, **i**. Black dots are σ-holes; the electrostatic potential is in kJ/mol. Color codes: gray, carbon; whitish, hydrogen; red, oxygen; blue, nitrogen; ocher, selenium.

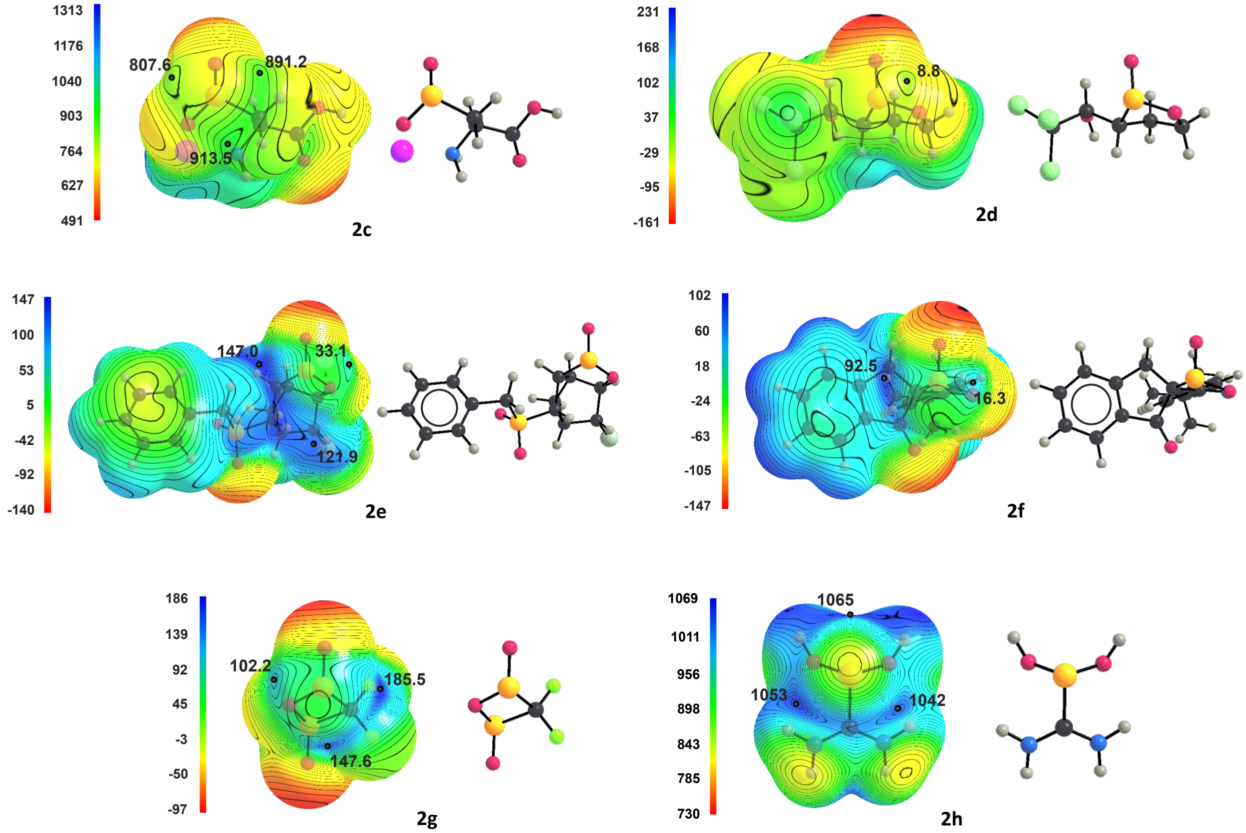

Figure 3S. Computed electrostatic potential on the 0.001 au molecular surfaces of sulfinic acid derivatives **2c**-**h**. Black dots are σ-holes; the electrostatic potential is in kJ/mol. Color codes: gray, carbon; whitish, hydrogen; red, oxygen; blue, nitrogen; ocher, selenium; violet cobalt.

3. Characterization of 1a

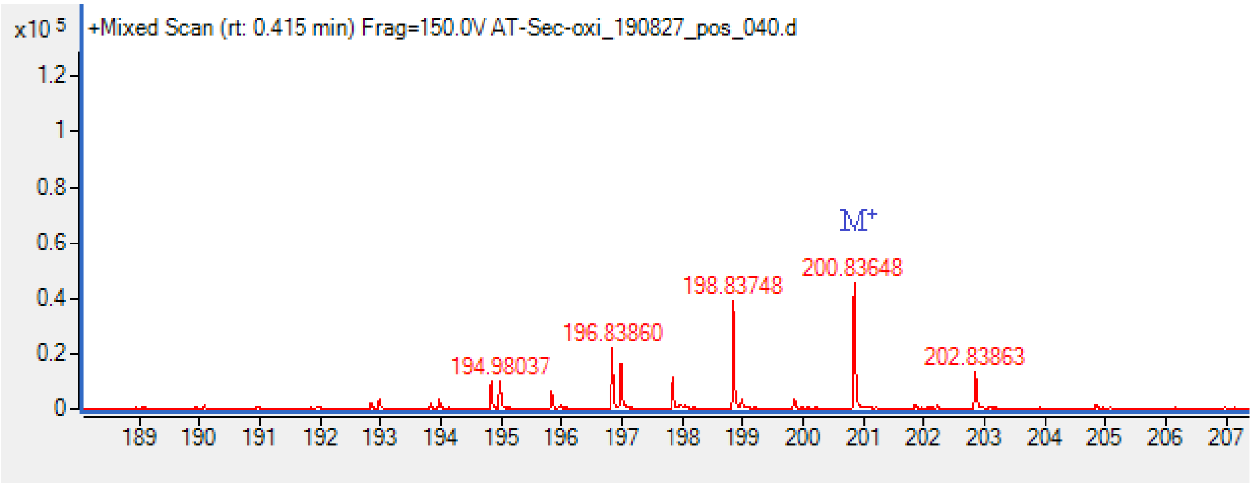

Figure 4S. HR-MS spectrum of compound **1a**.

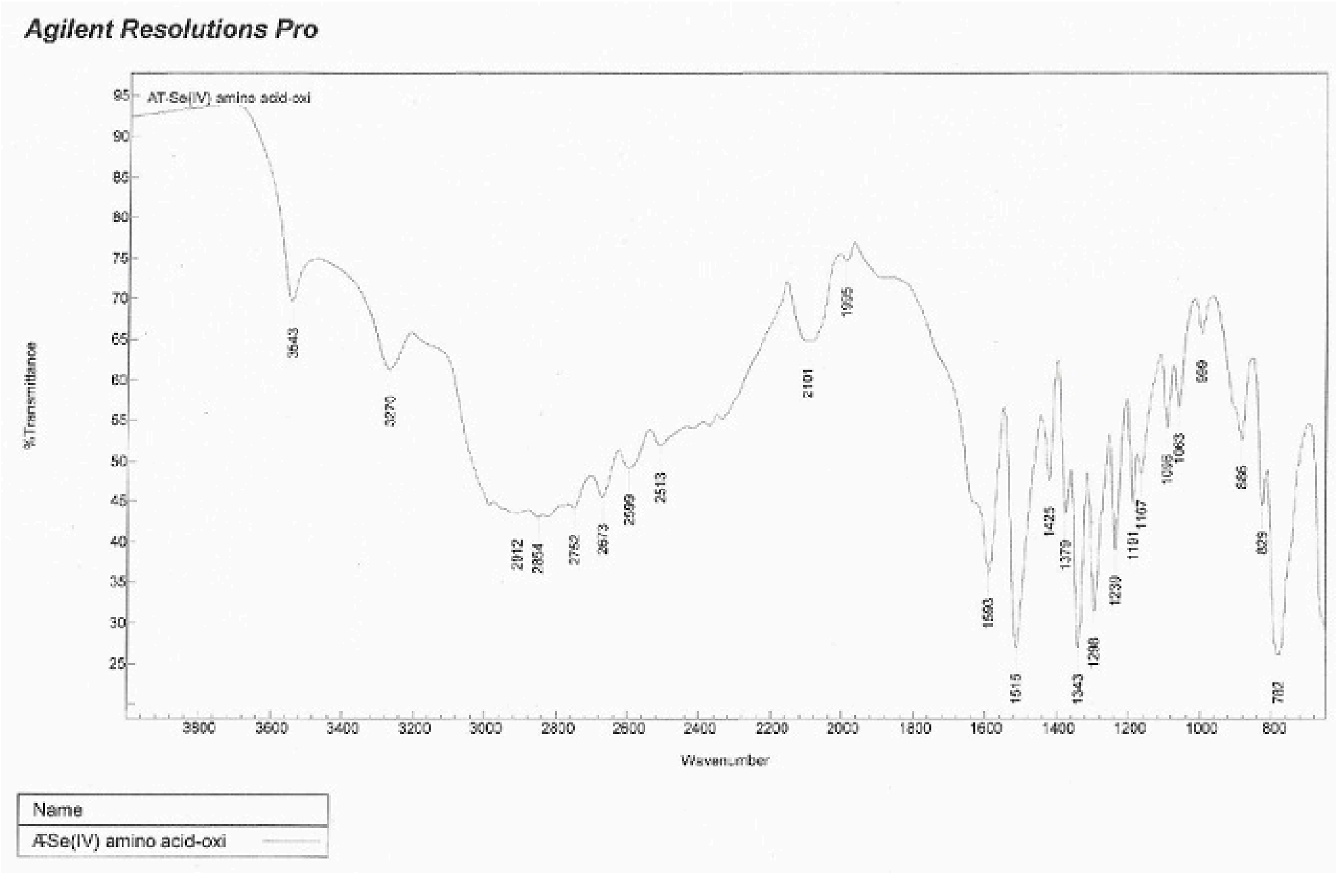

Figure 5S. IR spectrum of compound **1a**.

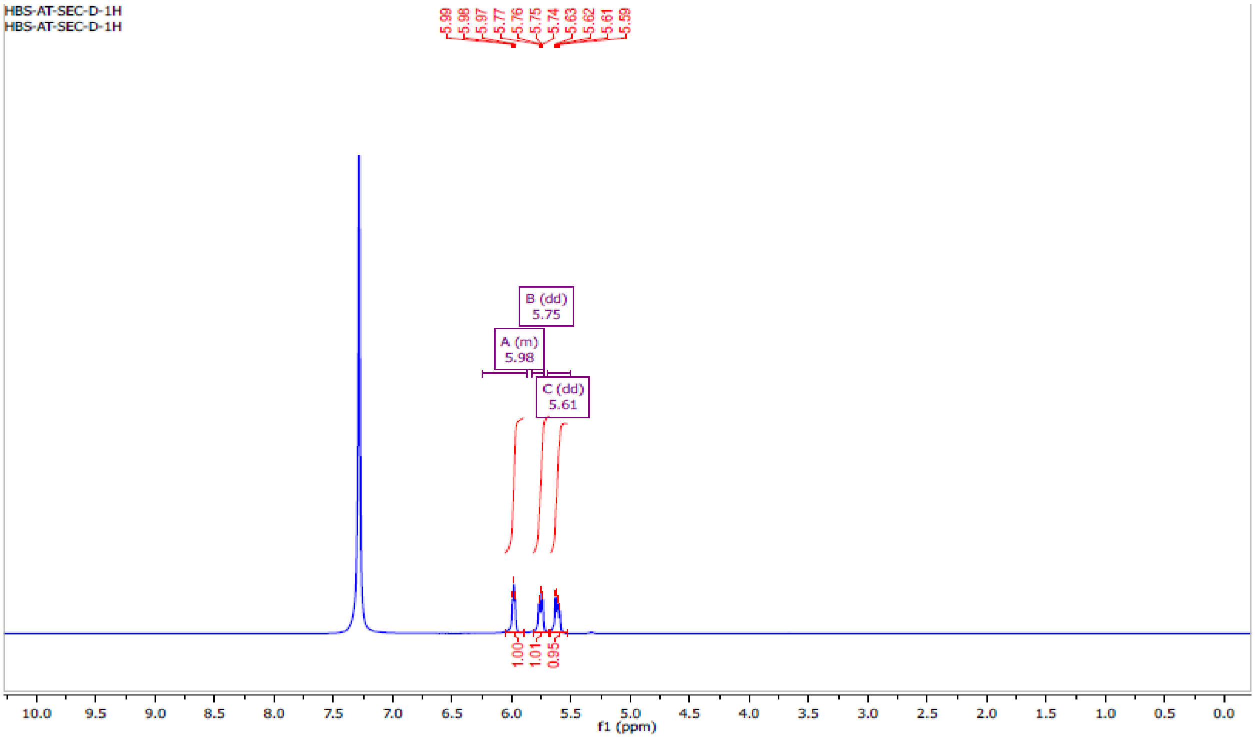

Figure 6S. ^{1}H NMR spectrum of compound **1a**.

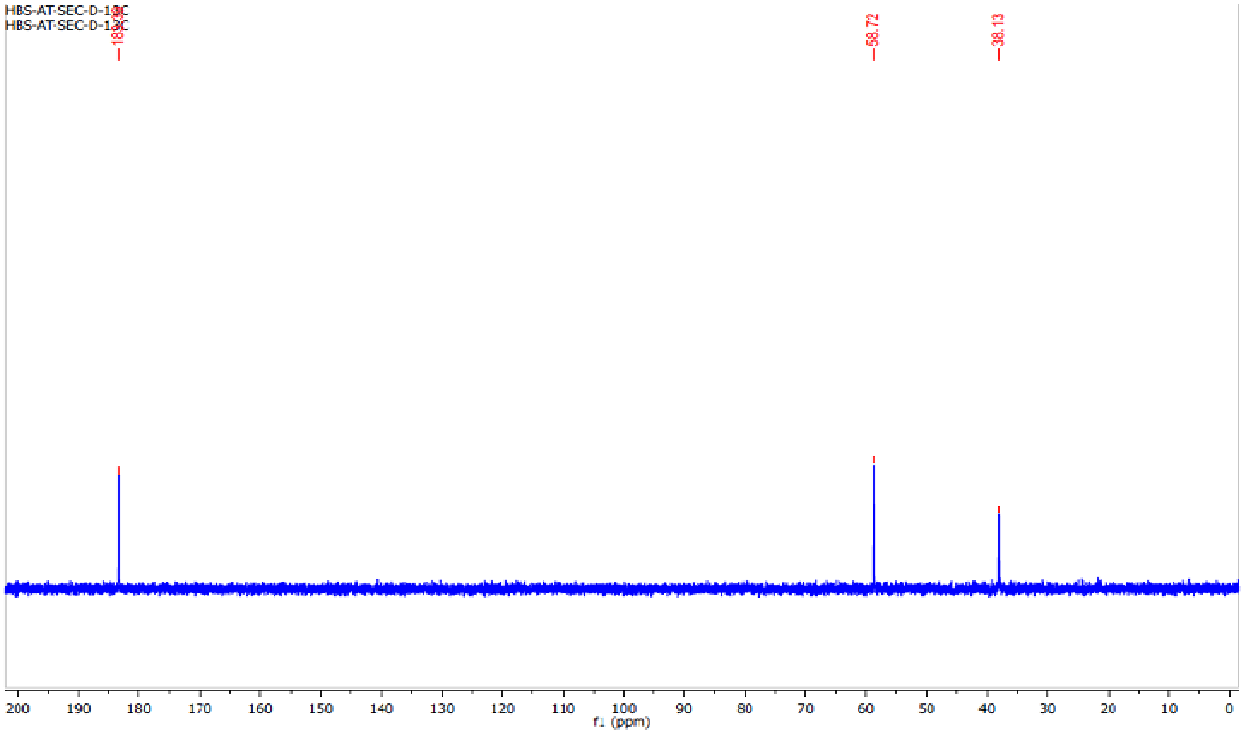

Figure 7S. ^{13}C NMR spectrum of compound **1a**.

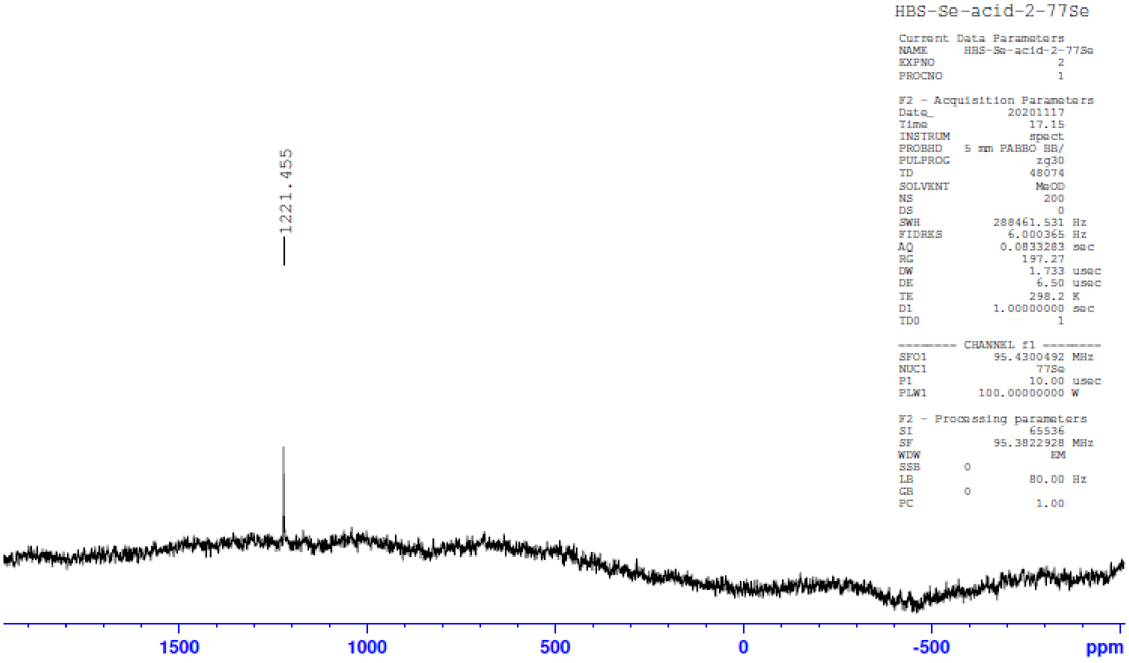

Figure 8S. ^{77}Se NMR spectrum of compound **1a**.

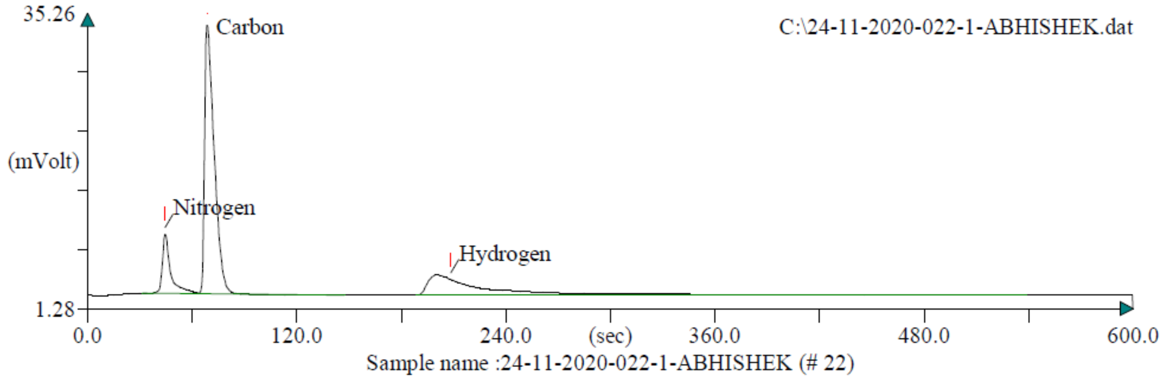

Peak Number (#)	Retention Time (min)	Area (.1*uV*sec)	Element %	Component
1	0.742	379533	6.007	Nitrogen
2	1.150	1961030	18.671	Carbon
3	3.475	886013	3.061	Hydrogen
		3226576	27.739	

Figure 9S. Elemental analysis of compound **1a**.

4. Crystallographic Details

Table 5S. Crystal data and structure refinement for **1a.**

Identification code	**1a**
Empirical formula	$C_3H_9NO_5Se$
Formula weight	218.07
Temperature/K	122.99(10)
Crystal system	orthorhombic
Space group	$P2_12_12_1$
a/Å	5.93050(10)
b/Å	10.72050(10)
c/Å	11.06300(10)
α/°	90
β/°	90
γ/°	90
Volume/Å^3	703.363(15)
Z	4
ρ_{calc}g/cm^3	2.059
μ/mm^{-1}	7.091
F(000)	432.0
Crystal size/mm^3	0.3 × 0.2 × 0.1
Radiation	CuKα (λ = 1.54184)
2Θ range for data collection/°	11.494 to 151.5
Index ranges	-4 ≤ h ≤ 7, -13 ≤ k ≤ 12, -13 ≤ l ≤ 13
Reflections collected	4056
Independent reflections	1318 [R_{int} = 0.0183, R_{sigma} = 0.0173]
Data/restraints/parameters	1318/0/99
Goodness-of-fit on F^2	1.115
Final R indexes [I>=2σ (I)]	R_1 = 0.0168, wR_2 = 0.0445
Final R indexes [all data]	R_1 = 0.0170, wR_2 = 0.0445
Largest diff. peak/hole / e Å^{-3}	0.40/-0.33
Flack parameter	-0.026(13)
CCDC number	2093715

Table 6S. Fractional Atomic Coordinates (×10^4) and Equivalent Isotropic Displacement Parameters (Å^2×10^3) for **1a**. U_{eq} is defined as 1/3 of of the trace of the orthogonalised U_{IJ} tensor.

Atom	*x*	*y*	*z*	U(eq)
Se1	-928.8(5)	4737.9(3)	6168.1(3)	11.24(11)
O5	6829(4)	2296(2)	7334(2)	22.1(6)
O4	-361(4)	4043(2)	9909(2)	19.5(5)
O3	-1243(4)	5306(2)	8369(2)	21.8(5)
O1	-376(4)	4077(2)	4716(2)	17.5(5)
O2	256(4)	6152(2)	6027(2)	14.6(5)
N1	3126(4)	3017(2)	8786(2)	12.7(5)
C1	1531(5)	3751(3)	6848(3)	13.4(7)
C3	-38(5)	4487(3)	8895(3)	14.1(6)
C2	2041(6)	4078(3)	8166(3)	12.6(6)

Table 7S. Anisotropic Displacement Parameters (Å^2×10^3) for **1a.** The Anisotropic displacement factor exponent takes the form: $-2\pi^2[h^2a^{*2}U_{11}+2hka^*b^*U_{12}+\ldots]$.

Atom	U_{11}	U_{22}	U_{33}	U_{23}	U_{13}	U_{12}
Se1	12.03(15)	11.81(16)	9.89(15)	1.70(13)	-0.57(14)	1.15(12)
O5	21.1(13)	22.3(13)	22.8(13)	-3.3(11)	-1.4(12)	6.3(11)
O4	25.8(14)	20.1(12)	12.7(11)	5.4(10)	6.8(10)	6.6(11)
O3	25.3(12)	24.1(12)	15.9(11)	6.6(11)	5.8(11)	14.6(12)
O1	18.3(13)	21.4(12)	12.8(12)	-1.4(10)	-3.5(10)	5.7(10)
O2	17.2(11)	12.8(10)	14.0(11)	3.0(9)	0.5(10)	-1.8(8)
N1	15.4(12)	13.1(12)	9.5(12)	-0.6(11)	0.1(12)	1.2(10)
C1	14.6(15)	15.7(15)	10.0(15)	1.3(12)	0.8(13)	4.5(13)
C3	17.3(14)	12.6(15)	12.5(15)	-1.1(13)	3.3(14)	-1.1(12)
C2	15.2(15)	12.5(14)	10.2(15)	3.2(12)	1.4(13)	1.2(13)

Table 8S. Bond lengths for **1a**.

Atom	Atom	Length/Å	Atom	Atom	Length/Å
Se1	O1	1.786(2)	O3	C3	1.273(4)
Se1	O2	1.678(2)	N1	C2	1.475(4)
Se1	C1	1.953(3)	C1	C2	1.530(4)
O4	C3	1.233(4)	C3	C2	1.537(4)

Table 9S. Bond angles for **1a**.

Atom	Atom	Atom	Angle/˚	Atom	Atom	Atom	Angle/˚
O1	Se1	C1	89.68(12)	O4	C3	C2	119.5(3)
O2	Se1	O1	101.40(12)	O3	C3	C2	114.1(3)
O2	Se1	C1	102.26(13)	N1	C2	C1	110.6(3)
C2	C1	Se1	113.0(2)	N1	C2	C3	109.0(3)
O4	C3	O3	126.5(3)	C1	C2	C3	114.0(3)

Table 10S. Hydrogen Atom Coordinates (Å×10^4) and Isotropic Displacement Parameters (Å^2×10^3) for **1a**.

Atom	*x*	*y*	*z*	U(eq)
H5A	7623.78	1744.11	7683.82	33
H5B	6912.39	2114.21	6587.42	33
H1A	4279.29	2744.44	8344.06	15
H1B	2127.62	2404.53	8879.73	15
H1C	3623.66	3261.69	9506.42	15
H1D	1143.08	2874.08	6796.62	16
H1E	2878.69	3884.08	6368.5	16
H2	3113.81	4773.48	8167.29	15
H1	-1530(90)	4250(50)	4320(40)	37(14)

5. References

1. Nakashima, Y.; Shimizu, T.; Hirabayashi, K.; Kamigata, N.; Yasui, M.; Nakazato, M.; Iwasaki, F. Isolation, absolute configuration, and chiral crystallization of optically active seleninic acid. *Tetrahedron Lett.* **2004**, *45*, 2301-2303.
2. Seff, K.; Heidner, E. G.; Meyers, M.; Trueblood, K.N. The crystal and molecular structure of methanesulfinic acid. *Acta Cryst.* **1969**, *B25*, 350-354.
3. Sarma, B. K.; Mugesh, G. Antioxidant activity of the anti-Inflammatory compound ebselen: a reversible cyclization pathway via selenenic and seleninic acid intermediates. *chem. Eur. J.* **2008**, *14*, 10603-10614.
4. Deka, R.; Sarkar, A.; Butcher, R. J.; Junk, P. C.; Turner, D. R.; Deacon, G. B.; Singh, H. B. Isolation of the novel example of a monomeric organotellurinic acid. *Dalton Trans.* **2020**, *49*, 1173-1180.
